# The Genetic Origin of the Indo-Europeans

**DOI:** 10.1038/s41586-024-08531-5

**Published:** 2025-02-05

**Authors:** Iosif Lazaridis, Nick Patterson, David Anthony, Leonid Vyazov, Romain Fournier, Harald Ringbauer, Inigo Olalde, Alexander A. Khokhlov, Egor P. Kitov, Natalia I. Shishlina, Sorin C. Ailincăi, Danila S. Agapov, Sergey A. Agapov, Elena Batieva, Baitanayev Bauyrzhan, Zsolt Bereczki, Alexandra Buzhilova, Piya Changmai, Andrey A. Chizhevsky, Ion Ciobanu, Mihai Constantinescu, Marietta Csanyi, Janos Dani, Peter K. Dashkovskiy, Sandor Evinger, Anatoly Faifert, Pavel Flegontov, Alin Frinculeasa, Mădălina N. Frinculeasa, Tamas Hajdu, Tom Higham, Paweł Jarosz, Pavol Jelinek, Valeri I. Khartanovich, Eduard N. Kirginekov, Viktoria Kiss, Alexandera Kitova, Alexeiy V. Kiyashko, Jovan Koledin, Arkady Korolev, Pavel Kosintsev, Gabriella Kulcsar, Pavel Kuznetsov, Rabadan Magomedov, Aslan M. Mamedov, Eszter Melis, Vyacheslav Moiseyev, Erika Molnar, Janet Monge, Octav Negrea, Nadezhda A. Nikolaeva, Mario Novak, Maria Ochir-Goryaeva, Gyorgy Palfi, Sergiu Popovici, Marina P. Rykun, Tatyana M. Savenkova, Vladimir P. Semibratov, Nikolai N. Seregin, Alena Šefčakova, Raikhan S. Mussayeva, Irina Shingiray, Vladimir N. Shirokov, Angela Simalcsik, Kendra Sirak, Konstantin N. Solodovnikov, Judit Tarnoki, Alexey A. Tishkin, Viktor Trifonov, Sergey Vasilyev, Ali Akbari, Esther S. Brielle, Kim Callan, Francesca Candilio, Olivia Cheronet, Elizabeth Curtis, Olga Flegontova, Lora Iliev, Aisling Kearns, Denise Keating, Ann Marie Lawson, Matthew Mah, Adam Micco, Megan Michel, Jonas Oppenheimer, Lijun Qiu, J. Noah Workman, Fatma Zalzala, Anna Szecsenyi-Nagy, Pier Francesco Palamara, Swapan Mallick, Nadin Rohland, Ron Pinhasi, David Reich

**Affiliations:** 1. Department of Human Evolutionary Biology, Harvard University, Cambridge, MA, USA.; 2. Department of Genetics, Harvard Medical School, Boston, MA, USA.; 3. Broad Institute of Harvard and MIT, Cambridge, MA, USA.; 4. Hartwick College, Department of Anthropology, Oneonta, NY, USA.; 5. Department of Biology and Ecology, Faculty of Science, University of Ostrava, Ostrava, Czechia.; 6. Department of Statistics, University of Oxford, Oxford, UK.; 7. Department of Archaeogenetics, Max Planck Institute for Evolutionary Anthropology, Leipzig, Germany.; 8. BIOMICs Research Group, Department of Zoology and Animal Cell Biology, University of the Basque Country UPV/EHU, Vitoria-Gasteiz, Spain.; 9. Ikerbasque-Basque Foundation of Science, Bilbao, Spain.; 10. Samara State University of Social Sciences and Education, Samara, Russia.; 11. Center of Human Ecology, Institute of Ethnology and Anthropology, Russian Academy of Science, Moscow, Russia.; 12. Department of Archaeology, State History Museum, Moscow, Russia.; 13. “Gavrilă Simion” Eco-Museum Research Institute, Tulcea, Romania.; 14. Historical Ecological and Cultural Association Povolzje, Samara Regional Public Organization, Samara, Russia.; 15. Azov History, Archaeology and Palaeontology Museum-Reserve, Azov, Russia.; 16. Institute of Archaeology named after A.Kh Margulan, Almaty, Kazakhstan.; 17. Department of Biological Anthropology, Institute of Biology, University of Szeged, Szeged, Hungary.; 18. Research Institute and Museum of Anthropology, Moscow, Russia.; 19. Institute of Archeology named after A. Kh. Khalikov Tatarstan Academy of Sciences, Kazan, Russia.; 20. Orheiul Vechi Cultural-Natural Reserve, Institute of Bioarchaeological and Ethnocultural Research, Chișinău, Republic of Moldova.; 21. Fr. I Rainer Institute of Anthropology, University of Bucharest, Bucharest, Romania.; 22. Damjanich János Museum, Szolnok, Hungary.; 23. Department of Archaeology, University of Szeged, Szeged, Hungary.; 24. Déri Museum, Debrecen, Hungary.; 25. Department of Regional Studies of Russia, National and State-Confessional Relations, Altai State University, Barnaul, Russia.; 26. Department of Anthropology, Hungarian Natural History Museum-Hungarian National Museum Public Collection Centre, Budapest, Hungary.; 27. Research Institute GAUK RO “Don Heritage”, Rostov-on-Don, Russia.; 28. Institute of Parasitology, Biology Centre of the Czech Academy of Sciences, České Budějovice, Czechia.; 29. Prahova County Museum of History and Archaeology, Ploiești, Romania.; 30. Department of Geography, Faculty of Humanities, University Valahia of Târgoviște, Târgovişte, Romania.; 31. Department of Biological Anthropology, Institute of Biology, Eötvös Loránd University, Budapest, Hungary.; 32. Department of Evolutionary Anthropology, University of Vienna, Vienna, Austria.; 33. Human Evolution and Archaeological Sciences, University of Vienna, Vienna, Austria.; 34. Department of Mountain and Highland Archaeology, Institute of Archaeology and Ethnology, Polish Academy of Science, Kraków, Poland.; 35. Slovak National Museum-Archaeological Museum, Bratislava, Slovak Republic.; 36. Peter the Great Museum of Anthropology and Ethnography, Department of Physical Anthropology, St. Petersburg, Russia.; 37. State Autonomous Cultural Institution of the Republic of Khakassia “Khakassian National Museum of Local Lore named after L.R. Kyzlasova”, Abakan, Russia.; 38. Institute of Archaeology, HUN-REN Research Centre for the Humanities, Budapest, Hungary.; 39. Centre for Egyptological Studies of the Russian Academy of Sciences, Russian Academy of Sciences, Moscow, Russia.; 40. Department of Archaeology and History of the Ancient World, Southern Federal University, Rostov-on-Don, Russia.; 41. Museum of Vojvodina, Novi Sad, Serbia.; 42. Department of History of the Institute of Humanities, Ural Federal University, Ekaterinburg, Russia.; 43. Institute of Plant and Animal Ecology, Urals Branch of the Russian Academy of Sciences, Yekaterinburg, Russia.; 44. Institute of History, Archaeology and Ethnography, Dagestan Branch of the Russian Academy of Science, Makhachkala, Dagestan, Russia.; 45. Independent researcher, Philadelphia, PA, USA.; 46. Department of General History, Historical and Literary Institute of the State University of Education, Ministry of Education Moscow, Moscow, Russia.; 47. Centre for Applied Bioanthropology, Institute for Anthropological Research, Zagreb, Croatia.; 48. Department of Archaeology and Heritage, Faculty of Humanities, University of Primorska, Koper, Slovenia.; 49. Kalmyk Scientific Centre of the Russian Academy of Sciences, Elista, Russia.; 50. National Agency for Archaeology, Chișinău, Republic of Moldova.; 51. National Research Tomsk State University, Tomsk, Russia.; 52. V.F. Voino-Yasenetsky Krasnoyarsk State Medical University, Krasnoyarsk, Russia.; 53. Department of Archaeology, Ethnography and Museology, Altai State University, Barnaul, Russia.; 54. Laboratory of Ancient and Medieval Archaeology of Eurasia, Altai State University, Barnaul, Russia.; 55. Slovak National Museum-Natural History Museum, Bratislava, Slovak Republic.; 56. Faculty of History, University of Oxford, Oxford, UK.; 57. Center for Stone Age Archeology, Institute of History and Archaeology, Ural Branch of the Russian Academy of Sciences, Ekaterinburg, Russia.; 58. Olga Necrasov Centre for Anthropological Research, Romanian Academy, Iași Branch, Iași, Romania.; 59. Tyumen Scientific Center of the Siberian Branch of Russian Academy of Sciences, Institute of Problems of Northern Development, Tyumen, Russia.; 60. Institute for the History of Material Culture, Russian Academy of Sciences, St Petersburg, Russia.; 61. Federal Research Centre “Fundamentals of Biotechnology” of the Russian Academy of Sciences, Moscow, Russia.; 62. Institute of Ethnology and Anthropology, Russian Academy of Sciences, Moscow, Russia.; 63. Howard Hughes Medical Institute, Harvard Medical School, Boston, MA, USA.; 64. Museo delle Civiltà, Italian Ministry of Culture, Rome, Italy.; 65. School of Archaeology, University College Dublin, Dublin, Ireland.; 66. Institute of Archaeogenomics, HUN-REN Research Centre for the Humanities, Budapest, Hungary.; 67. Wellcome Centre for Human Genetics, University of Oxford, Oxford, UK.; 68. These authors contributed equally: Iosif Lazaridis, Nick Patterson, David Anthony, Leonid Vyazov.

## Abstract

The Yamnaya archaeological complex appeared around 3300 BCE across the steppes north of the Black and Caspian Seas, and by 3000 BCE reached its maximal extent from Hungary in the west to Kazakhstan in the east. To localize Yamnaya origins among preceding Eneolithic people, we assembled ancient DNA from 428 individuals, demonstrating three genetic clines. A “Caucasus-Lower Volga” (CLV) Cline suffused with Caucasus hunter-gatherer^1^ ancestry extended between a Caucasus Neolithic southern end, and a northern end at Berezhnovka along the Lower Volga river. Bidirectional gene flow created intermediate populations, such as north Caucasus Maikop people, and those at Remontnoye on the steppe. The “Volga Cline” was formed as CLV people mixed with upriver populations of Eastern hunter-gatherer^2^ ancestry, creating hyper-variable groups as at Khvalynsk. The “Dnipro Cline” was formed as CLV people moved west, mixing with Ukraine Neolithic hunter-gatherers^3^ along the Dnipro river to establish Serednii Stih groups from whom Yamnaya ancestors formed around 4000 BCE and grew explosively after 3750–3350 BCE. CLV people contributed four-fifths of the ancestry of the Yamnaya, and, entering Anatolia likely from the east, at least a tenth of the ancestry of Bronze Age Central Anatolians, where Hittite was spoken^4,5^. We thus propose that the final unity of the speakers of “Proto-Indo-Anatolian”, the language ancestral to both Anatolian and Indo-European, was among CLV people sometime between 4400–4000 BCE.

## Introduction

Between 3300–1500 BCE, people of the Yamnaya archaeological complex and their descendants spread Indo-European languages from the steppe^2,6–12^ and transformed Europe, Central and South Asia, Siberia, and the Caucasus. Sparse sampling of both Yamnaya and their Eneolithic precursors poses a challenge for understanding the origins of this Bronze Age culture. It is broadly known that the Yamnaya had two ancestries: northern, “Eastern Hunter-Gatherer” (EHG) ancestry from far eastern Europe, and southern, West Asian ancestry^2^ from “Caucasus Hunter-Gatherers” (CHG) of Georgia,^1^ and Neolithic people from the Zagros^13^ and South Caucasus^10,14,15^. These two interacted across West Asia and eastern Europe,^13^ but where and how did the Eneolithic ancestors of the Yamnaya first appear? Potential northern ancestors include EHG, and EHG mixed with “Western Hunter-Gatherers” (WHG^16^) as in the Dnipro valley^3^ where they formed the Ukraine Neolithic hunter-gatherers (UNHG). But, the Yamnaya also received Anatolian Neolithic ancestry^9^, mediated via “Caucasus Neolithic” populations such as those sampled at Aknashen and Masis Blur of Armenia^10^ and even possibly Siberian ancestry that reached the European steppe before their emergence.^9^

We present a genetic analysis of 367 newly reported individuals (6400–2000 BCE) and increased data quality for 68 individuals^6^. The present study is the formal report for 291 and 63 of these; >80% are from Russia, and the rest largely from the western expansion into the Danube Valley (Supplementary Information section 1, Online Table 1). Details of 803 ancient DNA libraries (195 that failed screening) are in Online Table 2, and 198 new radiocarbon dates in Online Table 3. A parallel study^17^ of the North Pontic Region (Ukraine and Moldova) is the formal report for the remaining individuals. We labelled individuals based on geographical and temporal information, archaeological context, and genetic clustering (Online Table 4). The combined dataset adds 79 Eneolithic people from the European steppe and its environs to 82 published. It also adds 211 Yamnaya (and related Afanasievo) individuals to 75 published (Methods).

### Three Pre-Bronze Age genetic clines

**Figure 1:**
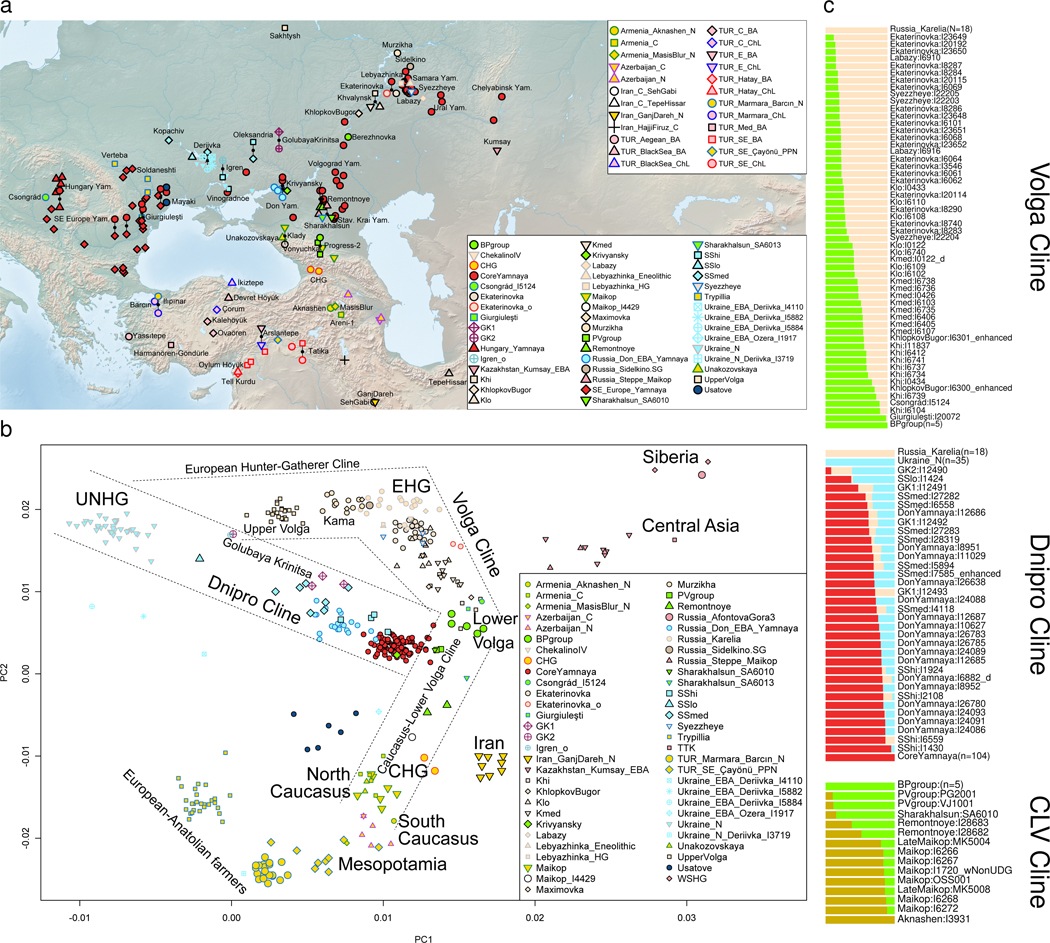
Three Eneolithic clines and their neighbors in space and time. (a) Map with analyzed sites. (b) PCA analysis using axes formed by a set of ancient West European hunter-gatherer (WHG), Siberian, West Asian, and European farmer populations. Selected individuals relevant to this study are projected (Methods). (c) qpAdm models fitted on individuals of the populations of the clines. The Volga Cline is generated by admixture between Lower Volga (BPgroup) people with upriver Eastern hunter-gatherers (EHG). People of the Dnipro Cline have UNHG or UNHG+EHG admixture relative to the Core Yamnaya (the hunter-gatherer source along this cline is significantly variable). The Caucasus-Lower Volga Cline is generated by admixture of lower Volga people with those from the Neolithic Caucasus (Aknashen-related).

Principal Component Analysis (PCA) of ancient individuals from the Pontic-Caspian steppe and adjacent areas reveals that Eneolithic people and the Bronze Age Yamnaya fall on non-overlapping gradients (Figure 1, Online Table 5). PC1 correlates (right to left) to differentiation between inland West Asian (Caucasus and Iran) and East Mediterranean populations (Anatolian-European)^14^, but interpretation is not clear as this axis also correlates to differentiation between Siberian and European hunter-gatherers. PC2 differentiates between northern Eurasians (top; including Europe and Siberia) and West Asians (bottom: Anatolia-Mesopotamia-Caucasus-Iran). Eneolithic and Bronze Age people occupy the middle, suggesting they formed by mixtures.

To distinguish alternative mixture scenarios that could explain these patterns, we implemented a competition framework around qpWave/qpAdm^2,18^ (Methods; Supplementary Information, section 2). The idea is that model *X* (a set of admixing sources) describes a target population *T* if it: (i) reconstructs the shared genetic drift of *T* with both distant outgroup populations and the sources of alternative models, but also (ii) renders these models infeasible if they cannot model shared drift with the sources of *X*. Models are thus first filtered against a set of distant outgroups; having survived this step, they are compared all-against-all to produce a set of promising models.

Three PCA clines (denoted geographically as “Volga”, “Dnipro”, and “Caucasus-Lower Volga”), diverge from the area enclosed by the Lower Don (at Krivyansky), Lower Volga (at Berezhnovka-2), and north Caucasus (at Progress-2, Vonyuchka-1, and Sharakhalsun^9^). They extend from there towards: (i) EHG and UNHG representing the pre-Eneolithic people of the Volga-Don-Dnipro area of eastern Europe, and (ii) CHG and Caucasus Neolithic representing the pre-Eneolithic people of the Caucasus and West Asia.

#### Volga Cline:

Distinct “upriver” and “downriver” gradients formed by Eneolithic individuals who lived on waterways that drain into the Caspian Sea delineate zones of ongoing human contact. PCA positions correlate well to positions along the Volga: the Volosovo-attributed Sakhtysh (in the Upper Volga) and Murzikha (near the Kama-Volga confluence)^19^ constitute the upriver “European Hunter-Gatherer Cline,” between EHG and UNHG. A “bend” separates the two clines and is occupied by EHG groups, including Middle Volga ones and those from northwest Russia in Karelia^2,20^, a very wide geographic distribution suggesting EHG was the earlier established population. Downriver and past the bend, we find the “Volga Cline”: hunter-gatherer affinity decreases at the Middle Volga at Labazy, Lebyazhinka, Ekaterinovka, Syezzheye, then Khvalynsk (4500–4350 BCE) and Khlopkov Bugor, before reaching the Lower Volga at Berezhnovka-2 (4450–3960 BCE) (Fig. 1a,b). This decrease is counterbalanced by increased affinity to the Caucasus, driven by an unsampled CHG-related source—somewhere between Georgia (the sampling location of CHG^1^) and the Lower Volga—interacting with EHG natives. Archaeological correlates for such interactions begin with the Seroglazovo forager culture expansion around the Lower Volga estuary ~6200 BCE, which parallels cultures of the Caucasus in ceramics and lithics, and continue to the North Caucasus Neolithic cemetery near Nalchik ~4800 BCE.^21,22^

At the end of the Volga Cline, four Lower Volga individuals from Berezhnovka-2 can be grouped with the north Caucasus PG2004 individual from Progress-2^9^ (4240–4047 BCE) into a “Berezhnovka-2-Progress-2 cluster” (“BPgroup”). The second Progress-2 individual (PG2001; 4994–4802 calBCE BCE) groups with another north Caucasus individual from Vonyuchka-1^9^ (VJ1001; 4337–4177 BCE) into a “Progress-2-Vonyuchka-1 cluster” (“PVgroup”). BPgroup and PVgroup are distinct (p=0.0006), but little differentiated (F_ST_=-0.002±0.002; Extended Data Table 1) suggesting movement between the north Caucasus piedmont and Lower Volga. The two locations also shared a distinctive burial pose on the back with raised knees, later typical of Yamnaya and dated earliest in four individuals from Ekaterinovka (4800–4500 BCE) contrasting with 95% of the graves posed supine with legs extended straight; and a female (individual #2) from Lebyazhinka-5, grave 12 (4838–4612 BCE). BPgroup is shifted relative to PVgroup (Fig. 1b), towards Afontova Gora-3 from Upper Paleolithic Siberia,^23^ West Siberian hunter-gatherers,^8^ and a 7,500-year old Neolithic individual from Tutkaul (TTK) from Central Asia.^20^

A natural interpretation is that upriver, EHG-related, and downriver, Berezhnovka-related, ancestors came together along the Volga, forming the genetic gradient. The upriver ancestry has long established eastern European antecedents,^20^ unlike the downriver ancestry, as (i) there are no earlier sequenced individuals from the Lower Volga, (ii) the Berezhnovka people are distinct from preceding groups, and (iii) BPgroup cannot be modeled as a clade with contemporary or earlier groups (p<0.001). Whatever BPgroup’s origins, we can use it as one proximate source for the Volga Cline together with an EHG source from Karelia^2,20^ well outside the Volga area and thus unlikely to be part of the riverine mating network. Seven Volga Cline populations fit this model (p-values of 0.04 for Ekaterinovka, 0.12–0.72 for the rest) with consistently poor fits only for Upper Volga, Murzikha, Maximovka, and “Klo” (the Khvalynsk individuals with low Berezhnovka relatedness) (p-values of 1e-66 to 0.006). Three of these (other than Klo) are arrayed in the upriver “European Hunter-Gatherer Cline” (Fig. 1c).

People buried at Ekaterinovka (5050–4450 BCE; based on three herbivore bone radiocarbon dates unaffected by marine reservoir effects; Online Table 1) were already mixing with Lower Volga Berezhnovka-related people (24.3±1.3%). This contrasts to the earlier hunter-gatherer from Lebyazhinka ( 7.9±3.6%; consistent with zero, p=0.21). A century or two later at Khvalynsk^24^, ~120km from Ekaterinovka (4500–4350 BCE; based on two herbivore bones), we observe an admixture gradient, divided for convenience into: “Khavlynsk high (Khi)” (76.8±1.9% BPgroup), “medium (Kmed)” (57.3±1.7% BPgroup), and “Khalynsk low (Klo)” (41.2±1.6% BPgroup). Volga Cline individuals had ~14–89% Berezhnovka ancestry (Fig. 1c), dominated by neither the old EHG natives nor the Lower Volga newcomers. Genetic differentiation between Lower Volga (BPgroup) and Ekaterinovka was strong (F_ST_=0.030±0.001; Extended Data Table 1), quite probably reflecting different linguistic-cultural communities.

A genetically Volga Cline individual from Csongrád-Kettőshalom in Hungary (4331–4073 BCE) had 87.9±3.5% BPgroup ancestry (Fig. 1c), comparable to “Khvalynsk high” individuals. This individual was from late 5^th^ millennium BCE steppe-like graves in Southeastern Europe that included a cemetery at Mayaky, Ukraine,^25–27^ and a cemetery at Giurgiuleşti,^28^ Moldova, from which one individual (I20072; 4330–4058 BCE) is a clade with BPgroup (p=0.90). Archaeology has documented Balkan copper on the Volga-Cline site of Khvalynsk,^24^ and the Csongrád and Giurgiuleşti individuals were plausibly part of this cultural exchange, leapfrogging the intervening Dnipro and Don basins without picking up ancestry from them.

#### (2) Dnipro Cline:

The Dnipro Cline is formed by Neolithic individuals living along the Dnipro River rapids (UNHG) (6242–4542 BCE) and the Serednii Stih population represented by 13 individuals (4996–3372 BCE; uncorrected for freshwater reservoir effects). This cline also includes most later Yamnaya individuals, a high quality and genetically homogeneous subset of which (n=104) we term “Core Yamnaya” (Supplementary Information, section 2). Close to Core Yamnaya (Fig. 1b) are some Eneolithic individuals: the Serednii Stih individual from Krivyansky in the Lower Don (4359–4251 BCE), and the PVgroup from the north Caucasus. Nonetheless, the Core Yamnaya cannot be modeled as derived from them or any other single source (p<1e-4). Dnipro Cline people are also distinct from Volga Cline ones, as no inter-riverine pairs form a clade (p<1e-7). This distinctiveness spans three millennia, commencing with the UNHG, continuing with the Eneolithic Serednii Stih, and ending with the Early Bronze Age Yamnaya. A geographically localized Yamnaya population of the Lower Don (n=23), many (n=17) of which are from the site of Krivyansky, are distinct from the Eneolithic individual at Krivyansky (Fig. 1b) and not a clade with them (p=8e-15). The Yamnaya can thus be traced neither to the north Caucasus (PVgroup), nor to the Lower Don (Krivyansky), nor to the Volga (BPgroup and the rest of the Volga Cline). Their placement on the Dnipro cline suggests their formation by a process of admixture as Serednii Stih culture descendants.

Serednii Stih heterogeneity contrasts with Core Yamnaya homogeneity (Fig. 1b), made more remarkable by the 5000 km-wide sampling of the latter, from Hungary to southern Siberia. The Yamnaya expanded across this vast region, hardly admixing with locals, at least initially and for elite individuals buried in kurgans. Serednii Stih culture individuals are arrayed along the Dnipro Cline. An individual from Vinogradnoe, grouped with two from Oleksandria and one from Igren, fall into an “SShi” cluster of greatest Core Yamnaya affinity but are not a clade with them (p=2×10^-7^). A Kopachiv female (I7585)^26^ is part of a “SSmed” cluster further along the cline, which also includes three individuals from Oleksandria and three from Deriivka. SShi and SSmed are largely contiguous, but I1424 from Moliukhiv Bugor (“SSlo”) is apart, close to UNHG. Variation within the Serednii Stih plausibly included unsampled individuals in gaps along the cline, or beyond its sampled variation. The Don Yamnaya largely overlap the Serednii Stih, and at stratified sites of the Lower Don Konstantinovka culture, they continued to occupy Serednii Stih settlements, a continuity unobserved in the Volga-Ural steppes.

All Dnipro Cline groups can be well modeled with either UNHG or GK2 (individual I12490 from Golubaya Krinitsa in the Middle Don; 5610–5390 BCE) at one extreme, and Core Yamnaya on the other (p-values 0.07 to 0.85). However, the hunter-gatherer end of the cline is not clearly one or the other: while the source for SSmed upriver fits equally well as UNHG (p=0.27) or GK2 (p=0.43), the Don Yamnaya upriver source can only fit as UNHG (p=0.08) not GK2 (p=0.0001), while the SShi upriver source can only fit as GK2 (p=0.08) not UNHG (p=0.003). We therefore model individuals from any point along the entire UNHG-EHG cline (Fig. 1c), not presupposing either UNHG or GK2 as the source, finding that UNHG ancestry predominates but additional EHG ancestry is also present (as at GK2). The hunter-gatherer source was thus from the Dnipro-Don (UNHG-GK2), not the Volga (EHG). GK2 clusters with Mesolithic hunter-gatherers from Vasylivka in the Dnipro^17^ and may stand in for unsampled survivors there of that earlier population. Core Yamnaya as a source for earlier populations is, of course, ahistorical: it must stand for an unsampled Eneolithic source.

**Figure 2.**
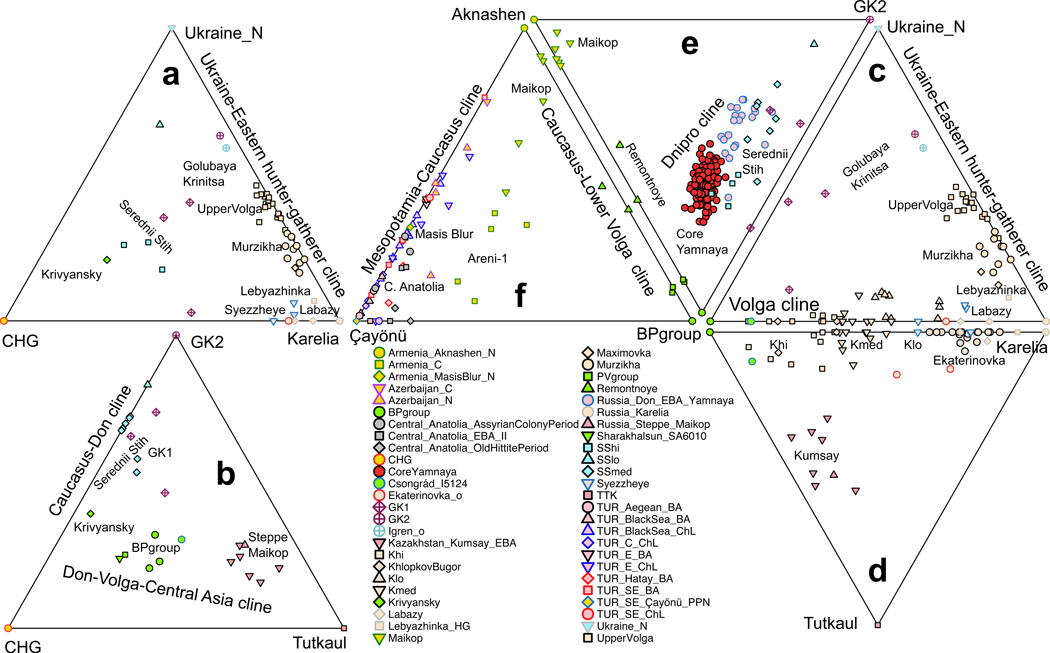
The three clines in the context of Eneolithic and Bronze Age admixture. Six 3-source qpAdm models elucidate a complex history of admixture. (a) Caucasus and European hunter-gatherer admixtures in the “Old Steppe”: Krivyansky on the Lower Don received much more CHG-related admixture than upriver people of the Middle Don at Golubaya Krinitsa. In the Middle and Upper Volga and the Kama River, populations had negligible CHG-related influence. (b) The “Don-Volga” difference. On the Lower Volga and North Caucasus piedmont, the BPgroup received CHG-related ancestry like its western Lower Don counterpart at Krivyansky; but, it also received ancestry from Central Asia; this eastern influence was higher still in the Bronze Age Steppe Maikop. (c) The Volga basin Eneolithic populations vis-à-vis the Don: populations at Khvalynsk, Klopkov Bugor, and Ekaterinovka form a Volga Cline between the Berezhnvoka cluster on the Lower Volga and the upriver EHG-like populations of the Middle Volga (Labazy and Lebyazhinka). (d) the Volga basin Eneolithic populations vis-à-vis Central Asia: a slight excess of Central Asian ancestry in the Khi subset of Khvalynsk. (e) the “Dnipro” cline: the Core Yamnaya are on one end of a cline, that also includes the Don Yamnaya and Serednii Stih populations, formed by admixture from the “Caucasus-Lower Volga” (CLV) cline of differential admixture of Neolithic Caucasus and BPgroup people. The CLV Cline includes diverse people buried in kurgans at Berezhnovka, Progress-2, Remontnoye, and Maikop sites Klady and Dlinnaya-Polyana ~5000–3000 BCE. (f) “West Asian”: CLV ancestry first appears in the Chalcolithic population at Areni-1 in Armenia and is also present in the Bronze Age at Maikop. The majority of the ancestry is from West Asian sources from the Mesopotamia-Caucasus (or Çayönü-Masis Blur-Aknashen) cline. Chalcolithic and Bronze Age Anatolians lack CLV ancestry but traces of it can be found in Bronze Age Central Anatolians.

The Don, between the Dnipro and Volga, is represented by Middle Don Golubaya Krinitsa individuals and the Lower Don Krivyansky. Golubaya Krinitsa contained archaeologically contrasting graves, one similar to those of the Dnipro Neolithic and the other to Serednii Stih.^29^ GK2 is modeled as 66.6±4.7% UNHG and 33.4±4.7% EHG (p=0.39). Using the most ancient sources (Karelia, UNHG, and CHG), Krivyansky Eneolithic and Golubaya Krinitsa individuals have variable CHG-related ancestry (Fig. 2a), maximized at Krivyansky (58.9±2.4%) and less (25.3±2.1%) in three Golubaya Krinitsa individuals grouped as GK1 (Fig. 1); GK2 had none or little (4.0±2.2%). Thus, the admixture history of the Don paralleled its intermediate geography, and included southern, CHG-related, ancestry (Fig. 2a). This was already present in GK1 (individual I12491/5557–5381 BCE)^11^ suggestive of an early presence, but its absence in GK2 of similar age proves that it was not generally present. GK1/GK2 dates may be inflated as Golubaya Krinitsa was archaeologically interpreted as being in cultural contact with the much later Eneolithic Serednii Stih.^30^ Moreover, a Serednii Stih outlier from Igren (I27930; 4337–4063 cal BCE) is a clade with GK2; this could be evidence of long-distance migration from the Don to the Dnipro in a Serednii Stih time frame. ^14^C dates at Golubaya Krinitsa could potentially be overestimated due to consumption of freshwater fish, which inflate dates by up to a millennium in this region.^31^

It has been suggested^11^ that the Yamnaya had ~35% CHG-related and ~65% Golubaya Krinitsa ancestry, the latter already having ~20–30% CHG-related ancestry, implying that the main Yamnaya source may have been hunter-gatherers of the Don area. Contradicting this model, Yamnaya do not fit models with CHG-related and either GK1/GK2 sources (p<10^-6^);^11^ to better understand this, we fit Yamnaya to a model of Karelia+UNHG+CHG (Fig. 2a), and found it underestimates shared drift of Core Yamnaya with both Afontova Gora-3 from Upper Paleolithic Siberia (Z=-5.2) and Anatolian Neolithic (Z=-6.8).^6^ A Volga source of the Siberian-related ancestry is suggested by the fact that applying the same model to Volga Cline groups also underestimates shared drift with Afontova Gora-3 (p=10^-8^ and Z=-4.5 for BPgroup; the Siberian ancestry is also evident in the deviation of the Dnipro cline towards Siberians in Fig. 1b). This Siberian-related ancestry is also affirmed as BPgroup can be modeled as ~76% Krivyansky and ~24% Central Asian (Siberian-related) Tutkaul^20^ (p=0.13). When we fit Krivyansky and BPgroup with the model that includes all relevant ancestries CHG, GK2, and Tutkaul (Fig. 2b), Krivyansky has little to no Central Asian ancestry (5.1±3.6%), fitting as a simple two-way mix of 56.7±2.6% CHG-related and 43.3±2.6% GK2 (p=0.37). In contrast, BPgroup requires 29.3±2.2% Tutkaul. Even adding Siberian-related ancestry (Tutkaul), however, is not sufficient to model the Core Yamnaya, as the three way of model of Fig. 2b still fails (p=10^-9^) to explain shared drift with Anatolian Neolithic (Z=-6.1).

“Central Asian” or “Siberian” ancestry was thus already in the North Caucasus steppe and Volga during the Neolithic, but with no evidence of it further west on the Don. Adding a third, western (UNHG) or eastern (Tutkaul), source (Fig. 2c,d) to the two-source BPgroup+EHG model for Volga Cline individuals, they remain well-modeled with these two alone (Fig. 2c). Some have more Tutkaul ancestry (Fig. 2d. However, deviations are minor (4.4±2.6% Tutkaul ancestry for “Khi”). Crucially, the Core Yamnaya fail all models of Fig. 2a-d (p<10^-8^): they were not formed of the CHG-EHG-UNHG-Tutkaul blend of these models.

#### (3) Caucasus-Lower Volga Cline (CLV):

The Core Yamnaya, being on the end of the Dnipro cline opposite that of the UNHG/GK2 (Fig. 1b), had ancestry from an unknown source of lower or even no such ancestry. The only consistently fitting (p=0.67) two-way model for them involved 73.7±3.4% of the SShi subset of Serednii Stih and 26.3±3.4% from a population represented by two Eneolithic individuals from Sukhaya Termista I (I28682) and Ulan IV (I28683) (4152–3637 BCE) near the village of Remontnoye, north of the Manych Depression between the Lower Don and Caspian Sea. Remontnoye is on neither the Volga nor Dnipro clines and does not form a clade (p<10^-10^) to any other group. It had at least two sources: a southern Caucasus one—either descendants of people like those who lived in Neolithic Armenia at Aknashen^10^, or ancestors of people of the Bronze Age Maikop^9^ culture—and a northern one from a population like BPgroup. The southern component can be modeled as having around half its ancestry from either Aknashen (44.6±2.7%; p=0.66) or Maikop (48.1±2.9%; p=0.44). We estimate -0.3±2.9% UNHG or -0.5±3.5% GK2 ancestry when either is added as a 3^rd^ source to the Aknashen+BPgroup model, so Remontnoye had no discernible UNHG/GK2-related ancestry as anticipated for the unknown source for the Yamnaya. Moreover, the main Maikop cluster, including individuals buried in kurgans in Klady and Dlinnaya-Polyana, had 86.2±2.9% (p=0.50) Aknashen ancestry. Thus, there exists a Caucasus-Lower Volga (CLV) cline: Aknashen-Maikop-Remontnoye-Berezhnovka. These four, arrayed in order of decreasing Caucasus Neolithic component, match their south-to-north location. North Caucasus people at Progress-2 and Vonyuchka-1 bucked the latitudinal trend, having, unlike their Maikop neighbors, little Caucasus Neolithic ancestry. These violations document long-range connectivity across the CLV area, and provide an important example of how genetics and geography do not always match.

Which group mediated the southern ancestry of the CLV cline? Not Aknashen, being geographically remote and much earlier (5985–5836 BCE). Not Maikop, which was geographically closer, but later (3932–2934 BCE). Unsampled Meshoko and Svobodnoe settlements (4466–3810 BCE)^32^ are plausible for the expansion of Aknashen-like ancestry northward and Berezhnovka-like ancestry southward, as they exchanged exotic stone, copper, and stone mace heads with Volga Cline sites. They are preceded in the North Caucasus by the Eneolithic Unakozovskaya (ref.^9^ 4607–4450 BCE, and this study) and succeeded by the Maikop. The Unakozovskaya population is not a good genetic source for Remontnoye, as the model BPgroup+Unakozovskaya fails (p<0.001) by overestimating (Z=3.8) CHG-related drift. Unakozovskaya is well modeled as 95.3±6.3% Maikop and 4.7±6.3% CHG (p=0.46); this group is therefore Maikop-like, but distinct genetically (p=2×10^-11^) (Fig. 1b). A recently published^33^ individual from Nalchik (c. 5000/4800 cal BCE) had more steppe affinity than the sampled Unakozovskaya, and can be modeled indeed (Supplementary Information section 2) as a mix of Unakozovskaya and steppe populations. Thus, in the Eneolithic North Caucasus there was: (i) Aknashen-related ancestry representing the Neolithic spread; (ii) CHG-related ancestry suggested by the Maikop-Unakozovskaya contrast; and (iii) northern Lower Volga ancestry constituting about one seventh the ancestry of sampled Maikop.

Remontnoye, Berezhnovka, and Maikop all employed kurgan burial, common 5000–3000 BCE in diverse CLV Cline people.^34^ By contrast, a distinctive burial position on the back with knees raised and the floor of the burial pit covered with red ochre was shared by all steppe groups including Serednii Stih, Volga Cline, and Remontnoye, while Maikop burials were contracted on one side. Some funeral customs united Maikop with the steppes, while others separated them.

The CLV Cline reveals that the ancestors of Dnipro Cline Serednii Stih and Yamnaya were CLV Cline people, similar to Remontnoye, who had been drawn into the Dnipro-Don region and mixed with locals. The actual sources for the Yamnaya may have differed from the sampled Remontnoye and SShi. The Dnipro Cline can be fit (Fig. 2e) by a 3-way model in which a Dnipro/Don hunter-gatherer source mixed with groups of mixed Aknashen and Berezhnovka ancestry. Either GK2 or UNHG can fit as the northern riverine source, but we use GK2 in Fig. 2e as this model has a higher p-value (p=0.93) than the UNHG alternative (p=0.04). The Yamnaya are inferred to have about a fifth of their ancestry from Dnipro/Don hunter-gatherers: either 22.5±1.8% GK2, or 17.7±1.3% or UNHG.

The CLV Cline was the vector by which Caucasus-derived ancestry flowed into the ancestors of the Yamnaya.^10^ The Remontnoye+SShi model predicts shared genetic drift with Neolithic Anatolians well (Z=-0.8), unlike models lacking Anatolian Neolithic ancestry (Fig. 2a-d). Archaeology has established that trade in Balkan copper during the late 5^th^ millennium BCE to North Caucasus farmer sites (Svobodnoe) and the Volga (Khvalynsk) took place, while Neolithic pots like those from Svobodnoe appeared in Dnipro-Don steppe sites connected with the Seredni Stih culture (Novodanilovka). This cultural exchange contextualizes the entry of BPgroup/Aknashen-mixed groups into the Dnipro-Don steppes.

#### CLV impact in the Armenia and Anatolia:

CLV Cline people also went south (Fig. 2f), explaining the steppe ancestry found at Areni-1 in Chalcolithic Armenia around 4000 BCE^13^, where Lower Volga ancestry (26.9±2.3% BPgroup) admixed with a local Masis Blur-related Neolithic substratum (Supplementary Information section 2). This contrasts with the North Caucasus Maikop where the substratum was Aknashen-related. We can model Masis Blur as 33.9±8.6% Aknashen and 66.1±8.6% Pre-Pottery Neolithic of the Tigris Basin of Mesopotamia^35^ at Çayönü (p=0.47), part of a Neolithic Çayönü-Masis Blur-Aknashen cline. The populations of Armenia retained CHG differentially^6^: more (42.0±3.8%) in Aknashen than in Masis Blur (13.7±4.0%). Some Anatolian Chalcolithic and Bronze Age groups can be derived entirely from the Caucasus-Mesopotamian cline (Fig. 2f), while others also have ancestry from the Mesopotamian-Anatolian cline, lacking any steppe ancestry.^10,34,36–38^

**Extended Data Figure 1:**
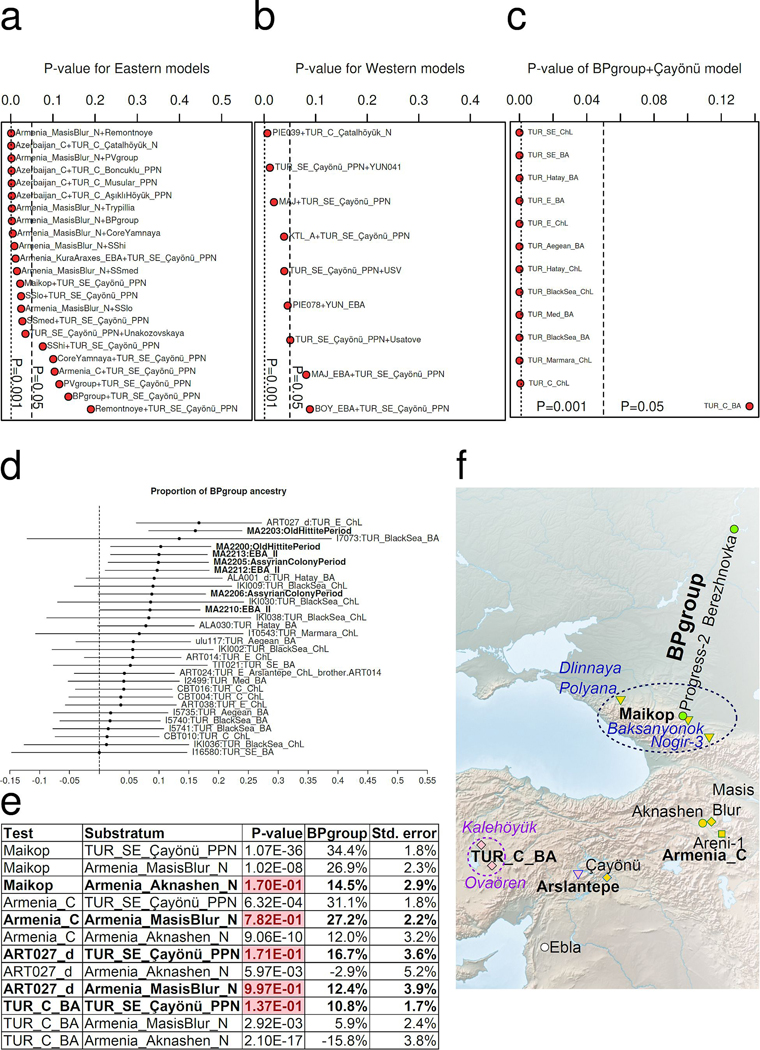
The origin of Central Anatolian Bronze Age people. (a) Models with eastern steppe sources (including CLV and Serednii Stih). Fitting models include Mesopotamian (Çayönü) and steppe ancestry. (b) Models with western sources, including Usatove and those from Southeastern Europe fail except those with Çayönü and either Mayaky or Boyanovo EBA (both of which are Yamnaya-derived). (c) The steppe (BPgroup)+Çayönü model fails all Chalcolithic/Bronze Anatolians except Central Anatolian Bronze Age. (d) Steppe (BPgroup) ancestry in the BPgroup+Çayönü model is observed in all individuals of the Central Anatolian Bronze Age (mean and ±3 s.e. estimated by qpAdm are shown for all Chalcolithic and Bronze Age individuals from Anatolia that fit the model at p>0.05) as well as in individual ART027_d from Chalcolithic Arslantepe in Eastern Anatolia. (e) BPgroup-related ancestry admixed with different substrata: Aknashen-related in the North Caucasus Maikop, Masis Blur-related in Chalcolithic Armenia, and Mesopotamian-related (Çayönü) in the ancestors of the Central Anatolian Bronze Age, following the route (f) from the North Caucasus to Anatolia; sites with BPgroup-related ancestry marked in bold. In all panels p-values estimated by qpWave are shown.

We show that Central Anatolians^34^ from the Early Bronze Age (2750–2500 BCE), Assyrian Colony (2000–1750 BCE), and Old Hittite (1750–1500 BCE) periods were unusual in the Anatolian landscape as they had CLV ancestry combined with Mesopotamian (Çayönü) (Supplementary Information, section 2; Fig. 2f; Extended Data Fig. 1). The non-Mesopotamian ancestry varied depending on the level of CLV “dilution” : 10.8±1.7% ancestry (p=0.14) from BPgroup, or 19.0±2.4% from Remontnoye (p=0.19), or 33.5±4.8% from Armenia_C (p=0.10).

The exact source of the steppe ancestry in Anatolia cannot be precisely determined, but all fitting models involve some of it (Extended Data Fig. 1a). Some of the steppe-related sources are unlikely on chronological-linguistic grounds; for example, the Core Yamnaya itself (12.2±2.0%; p=0.10) as well as western Yamnaya-derived populations from Southeastern Europe such as from Boyanovo or Mayaky Early Bronze Age^25^ (Extended Data Fig. 1b). The Early Bronze Age Central Anatolians from Ovaören^34^ (2750–2500 BCE) do temporally overlap the late Yamnaya period but the timing of the Yamnaya expansion is in tension with the much earlier linguistic split of Anatolian languages that form an outgroup to those of the inner Indo-European core.^39^ Fixing Çayönü as one source and adding pairs of steppe sources (allowing ancestry to range freely along the Volga, Dnipro, and CLV clines), the hunter-gatherer contribution is negative on the Volga Cline (-3.4±2.6% EHG), and on the Dnipro Cline (-2.3±2.7% UNHG or -3.9±3.5% GK2); thus, the admixing population had no more EHG/UNHG/GK2 ancestry than the BPgroup/Core Yamnaya endpoints of these two clines (Supplementary Information section 2). Placing the admixing population on the CLV cline is successful (p=0.129) with a significant amount of BPgroup ancestry (8.8±2.7%) validating a CLV and north-of-the-Caucasus mountains Eneolithic origin. Steppe+Mesopotamian models fit the Central Anatolian Bronze Age but none of the Chalcolithic/Bronze Age Anatolian regional subsets (p<0.001; the BPgroup+Çayönü model is shown in Extended Data Fig. 1c): their success is not due to their general applicability. Moreover, steppe ancestry in the Central Anatolian Bronze Age is observed across individuals and periods (Extended Data Fig. 1d), including Early Bronze Age Ovaören south of the Kızılırmak river and Middle/Late Bronze Age Kalehöyük just within the bend of the river. This is consistent with an Anatolian-Hattic linguistic boundary coinciding with the Kızılırmak, a boundary breached before the ca. 1730 BCE conquest of Hattusa by the Hittites.^4^ Regardless of the (inherently unknowable) linguistic identity of the sampled individuals, their unique blend of ancestries demands an explanation.

**Extended Data Figure 2:**
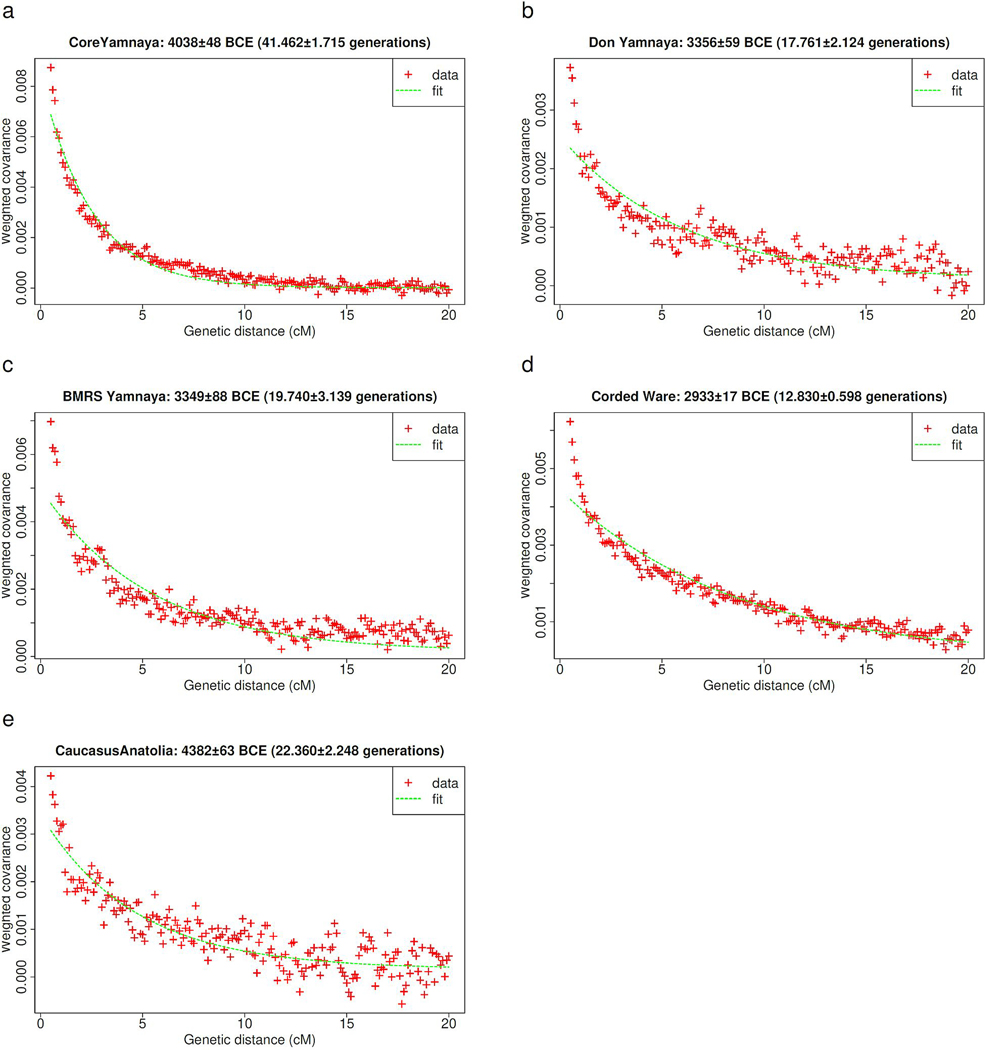
Admixture date estimates. We estimate admixture dates for the Core Yamnaya as a mixture of European hunter-gatherer and West Asian populations (a), for the Don Yamnaya as a mixture of Core Yamnaya and UNHG (b), for the Bulgaria, Moldova, Romania, and Serbia (BMRS) Yamnaya as a mixture of Core Yamnaya and European Neolithic/Chalcolithic farmers (c), for the Corded Ware as a mixture of Core Yamnaya and Globula Amphora (d), and for a combined Caucasus-Anatolia population (Maikop-Armenia_C-TUR_C_BA) a mixture of European hunter-gatherer and West Asian populations which occurred ca. 4400 BCE (e). The Core Yamnaya were formed ca. 4000 BCE, followed by admixture ca. 3350 BCE with UNHG and European farmers in the east and west of the Dnipro-Don region and <3000 BCE in central-eastern Europe.

Populations along the path to Central Anatolia can be modeled with BPgroup ancestry and distinctive Caucasus-Mesopotamian substrata: Aknashen-related in the North Caucasus Maikop; Masis Blur-related in Chalcolithic Armenia; and Mesopotamian Neolithic in the Central Anatolian Bronze Age (Extended Data Fig. 1e, f). These admixtures had begun by ca. 4300–4000 BCE (the date range of the Armenia_C population^13^) and we date them to 4382±63 BCE (Extended Data Fig. 2e). The Pre-Pottery Neolithic population of Çayönü was genetically halfway between that of Mardin^14^, 200km to the east, and the Central Anatolian pottery Neolithic at Çatalhöyük^40^ along the Mesopotamian-Anatolian cline. Chalcolithic/Bronze Age people from Southeastern and Central Anatolia all stemmed from the same Çatalhöyük-Mardin continuum, (Supplementary Information section 2). If the Proto-Anatolians came from the east, then their descendants may have been at the state of Armi whose precise location is uncertain but whose Anatolian personal names are recorded by their Kingdom of Ebla neighbors in Syria^5^ in the 25^th^ c. BCE, half a millennium before Anatolian languages are attested, and just south of the proposed migratory path (Extended Data Fig. 1f). We thus propose that CLV cline people migrated southwards ca. 4400 BCE, a millennium before the Yamnaya, admixed along the way, and finally reached Central Anatolia from the east.

**Figure 3.**
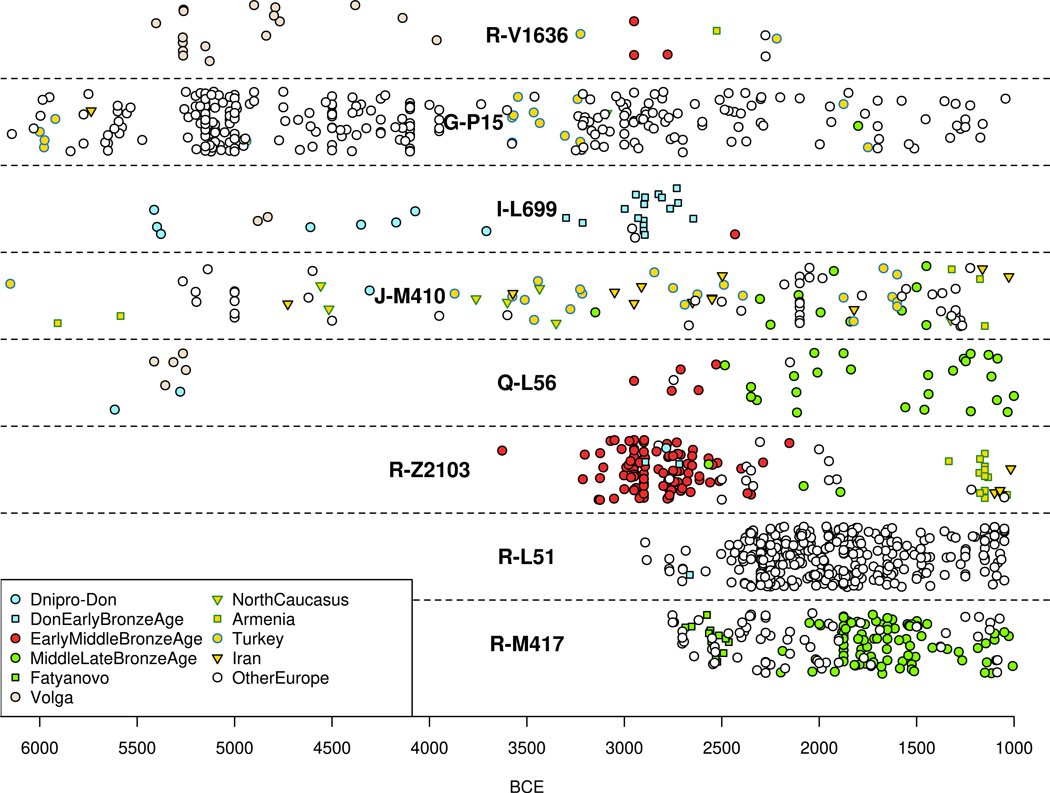
Patrilineal succession. Temporal distribution of key Y-chromosome haplogroups from Kazakhstan, Kyrgyzstan, Mongolia, Russia, Turkmenistan, Ukraine, Uzbekistan, and comparative regions of Europe and West Asia 6000–1000 BCE. The Early and Middle Bronze Age group includes the Yamnaya, Afanasievo, Poltavka, Catacomb, Chemurchek, and North Caucasus cultures; the Middle and Late Bronze Age group individuals of diverse cultures down to 1000 BCE including those of the Sintashta, Andronovo, Potapovka, and Srubnaya cultures. Information on which individuals are plotted can be found in Online Table 6.

We find Y-chromosome evidence consistent with this reconstruction: sporadic instances of steppe-associated Y-chromosome haplogroup R-V1636 in West Asia at Arslantepe^37^ in Eastern Anatolia and Kalavan^13^ in Armenia in the Early Bronze Age (~3300–2500 BCE) among individuals without detectible steppe ancestry.^10,13^ The R-V1636 individual (ART038) from Arslantepe does not provably have BPgroup ancestry (3.6±3.1%) but ART027 from the same site (3370–3100 BCE) does (16.7±3.5%, p=0.171), preceding the same mix in Early Bronze Age Central Anatolia by a few centuries. R-V1636 in the Remontnoye male, both from Progress-2^9^, two of three from Berezhnovka, and eleven individuals of the Volga Cline, prove it to be a prominent lineage of the pre-Yamnaya steppe, and it also appeared as far as northern Europe.^41,42^ A single R-V1636 individual (SA6010; 2886–2671 BCE) from Sharakhalsun,^9^ consistent with CLV ancestry (Fig. 2), is found post-Yamnaya, a holdout of this once pervasive lineage (Fig. 3).

### The Yamnaya expansion

**Extended Data Figure 3:**
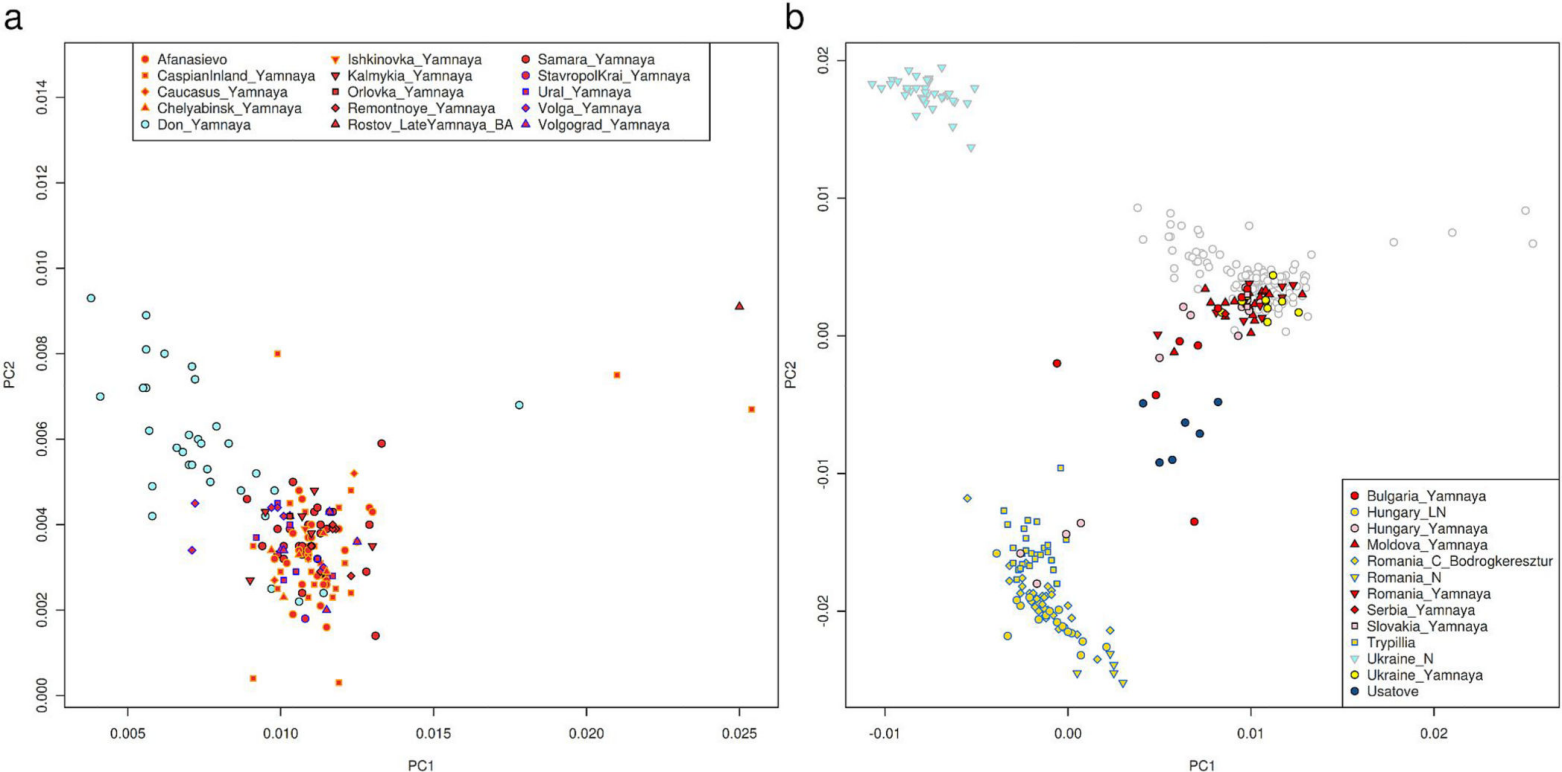
Population structure in people with a Yamnaya cultural affiliation. Individuals are projected in the same space as in Fig. 1. (a) showing that the Core Yamnaya cluster (red fill symbols) from diverse sites is differentiated from the Don Yamnaya (blue fill) who tend towards the UNHG. (b) Yamnaya individuals in the West (Ukraine, Hungary, Slovakia, and Southeastern Europe) include a tight cluster of individuals as well as others that tend towards the direction of European Neolithic and Chalcolithic groups from Romania and Hungary. Individuals from Russia are shown in grey circles in panel (b). Coordinates of plotted points can be found in Online Table 6.

We infer the average date of mixture in Core Yamnaya^43^ to be 4038±48 BCE (Extended Data Fig. 2a), with sources related to UNHG/EHG hunter-gatherers, and West Asian/Caucasus-related people (Fig. 1b). Such a date does not preclude the possibility that the mixture began before or continued afterward, but corresponds strikingly to the efflorescence of the Serednii Stih culture. The ancestors of Core Yamnaya (Extended Data Table 2) (Fig. 1b) must have been geographically constrained,^17^ contrasting with their later distribution from China to Hungary (Extended Data Table 2, Extended Data Fig. 3a, Online Table 6) even while maintaining high genetic similarity (mean F_ST_=0.005) (Extended Data Table 3). The Don Yamnaya (Extended Data Fig. 3a) are modeled as 79.4±1.1% Core Yamnaya and 20.6±1.1% UNHG. The non-Yamnaya component may be underestimated, if, as is plausible, the Core Yamnaya admixed with a Serednii Stih population of partial UNHG ancestry. We estimate that the Don Yamnaya formed in the late 4^th^ millennium BCE (Extended Data Fig. 2b), when, one may assume, unmixed UNHG were rare.

The western expansion also brought Yamnaya into southeastern Europe reaching as far as Albania and Bulgaria.^10^ Many of these cluster with the Core Yamnaya, but others deviate towards Neolithic and Chalcolithic populations of southeastern and central Europe (Extended Data Fig. 3b). Yamnaya admixture with these (Extended Data Table 4) occurred in the late 4^th^ millennium BCE (Extended Data Fig. 2c), after sporadic early Chalcolithic migrations into southeastern Europe from the steppe.^25^ By contrast, the Don Yamnaya expanded little, as virtually no individuals with high quality data outside the Don are a clade with them (Supplementary Information, section 2): the Lower Don was a cul-de-sac for the Yamnaya expansion.

Y chromosome haplogroup sharing is not informative for Core Yamnaya origins but proves that the Don Yamnaya, dominated by haplogroup I-L699 (17/20 instances), had continuity with their Serednii Stih and Neolithic hunter-gatherer ancestors (Fig. 3, Online Table 7). The Core Yamnaya belonged to R-M269 (49/51 instances) most of which was the R-Z2103 (41/51) sub-lineage, undetected before the Yamnaya period and related to R-L51, prevalent among Bell Beaker burials^7^ and non-steppe Europe (Fig. 3). Slightly more distant is R-PF7563, found in Mycenaean Greece. R-L23, formed ~4450 BCE (https://www.yfull.com/tree/R-L23/ ; v12.04.00), unifies in the Eneolithic Beakers, Yamnaya, and Mycenaeans. Population divergences are lower than haplogroup ones, so these lineages may have co-existed within the Yamnaya. Finding the R-L23 founder population remains challenging, but our failure to sample it to date it is not surprising if it was small and isolated.

**Extended Data Figure 4:**
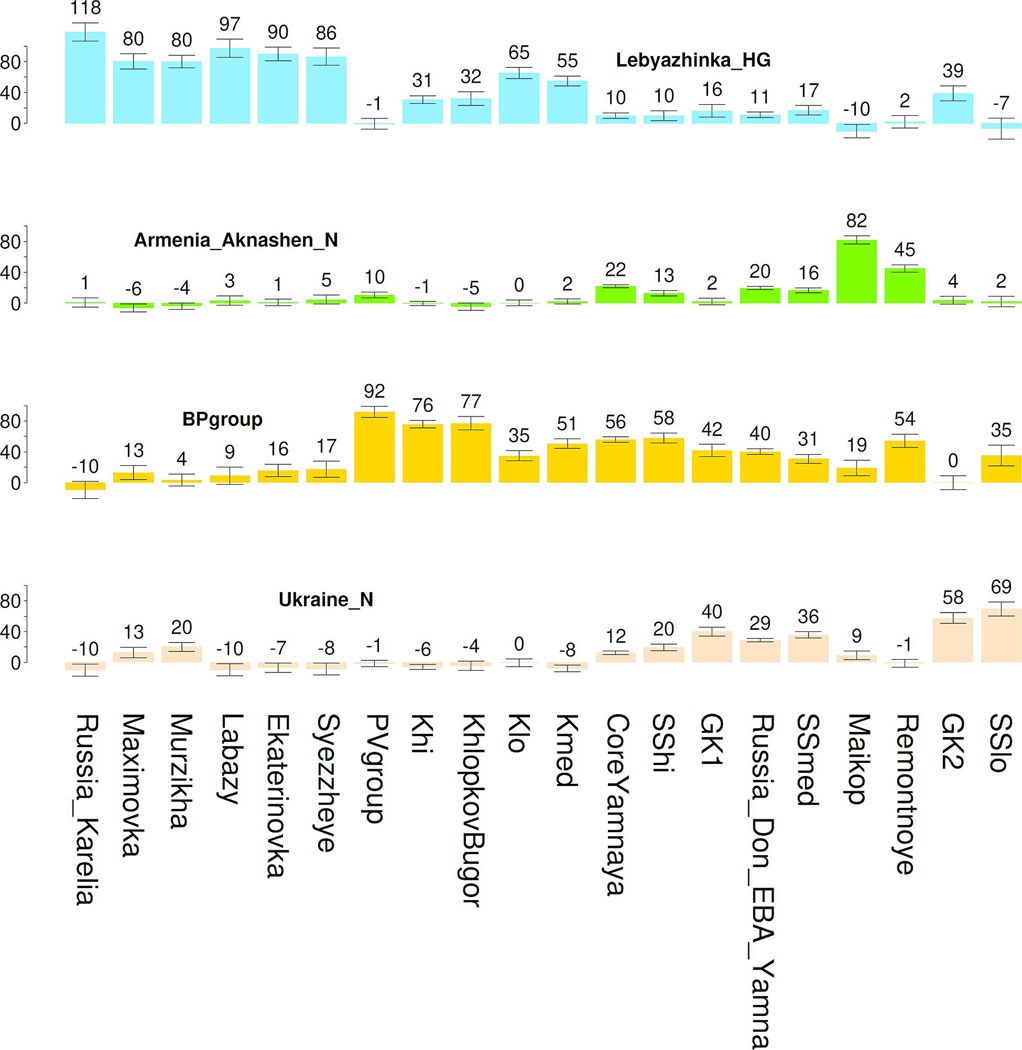
A 4-way model for the entire Dnipro-Don-Volga-Caucasus region. Mean and ±1 standard error estimated by qpAdm is shown.

That the Core Yamnaya are part of the Dnipro cline may suggest an origin in the Dnipro basin itself, but (a) the Dnipro cline is generated by admixture with Dnipro-Don people (UNHG/GK2-related), and (b) the Yamnaya on the Don are also part of this cline, so an alternative origin in the Don area cannot be excluded. Solutions further east are unlikely since the Yamnaya are on neither Volga nor CLV clines. Likewise for solutions west of the Dnipro: the Core Yamnaya have scant or no European farmer ancestry (from the west) (Fig. 1b).^17^ A more western origin of the Core Yamnaya would also bring their latest ancestors in proximity to the likely founders of the Corded Ware complex whose origin is itself in question but must have certainly been in the area of central-eastern Europe occupied by the Globular Amphora culture west of the Core Yamnaya. Most Corded Ware individuals, who can be fit as tracing a large part of their ancestry to the Yamnaya,^2,12^ were formed by admixture concurrent with the Yamnaya expansion^43^ (Extended Data Fig. 2d), shared segments of IBD proving genealogical timeframe connections,^44^ and had a balance of ancestral components for their non-European farmer-related ancestry indistinguishable from the Yamnaya.^6^ The early 3^rd^ millennium BCE history of the Corded Ware population is intertwined with the Yamnaya expansion as it involved admixture with genetically—if not necessarily archaeologically—Yamnaya people. The Dnipro-Don area of the Serednii Stih culture, fits the genetic data, as it explains the ancestry of the nascent Core Yamnaya. All ancestral components found in the Serednii Stih and lacking elsewhere are found in the Yamnaya (Extended Data Fig. 4), and from the Dnipro-Don area both Corded Ware, and Southeastern European Yamnaya (in the west) and the Don Yamnaya (in the east) could have emerged by admixture of the Core Yamnaya with European farmers and UNHG descendants respectively.

**Figure 4:**
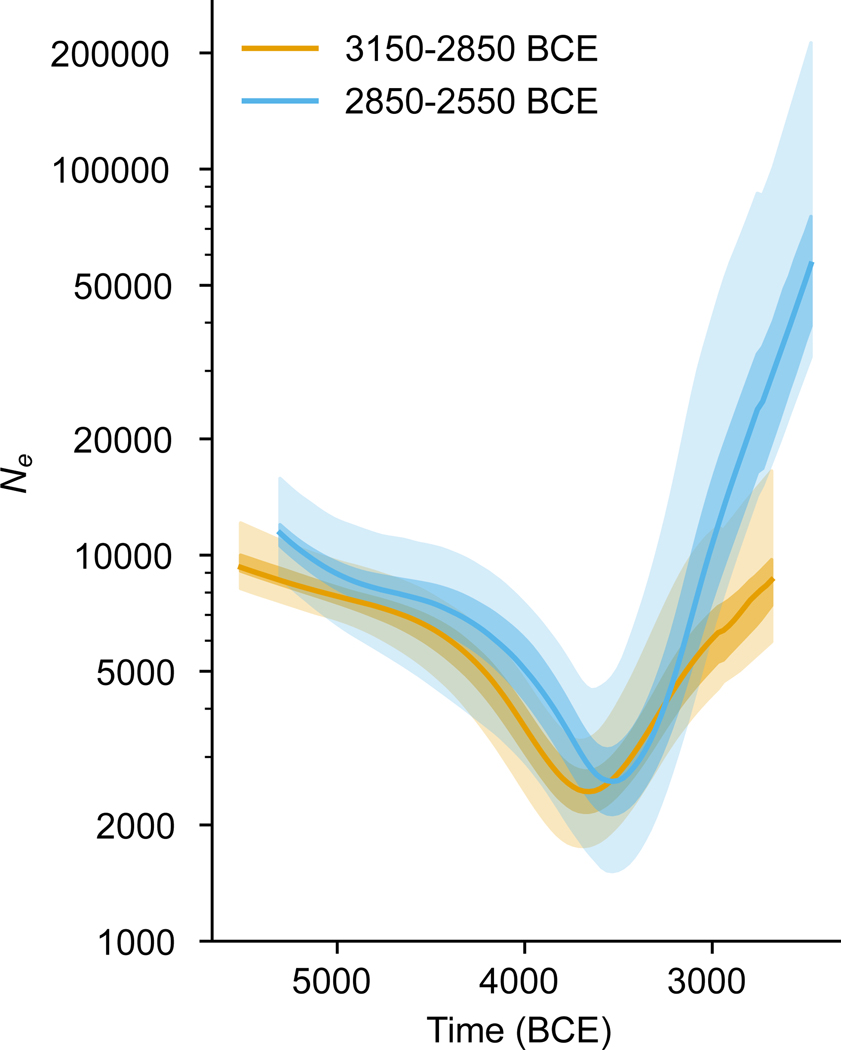
Trajectory of the Yamnaya expansion. We use HapNe-LD to estimate the changes in effective population size over time of Yamnaya ancestors, performing the computation separately for the individuals from the earlier three hundred years of our sampling, and the later three hundred years; shading shows confidence intervals (dark: 50%, light: 95%). Jointly displaying these two trajectories reveals an extraordinary population expansion after 3642–3374 BCE (intersection of 95% confidence intervals for the two analyses for the minimum), from when the effective size is a few thousand to an order of magnitude larger. The offset on the x-axis is due to the difference in sampling time between the two groups.

We estimated the population growth of Core Yamnaya using HapNe-LD, which infers effective population size fluctuations in low-coverage ancient DNA data.^45^ Core Yamnaya dating to the first three hundred (n=25) and later three hundred (n=26) years of our sampling produce 95% confidence intervals of 3829–3374 BCE and 3642–3145 BCE for the time before growth (Fig. 4). For both, these correspond to growth from an effective number of reproducing individuals of a few thousand. These intervals overlap at 3642–3374 BCE, the late Serednii Stih period. Taken together with the admixture dating, a scenario emerges where Yamnaya ancestors were formed by admixture around 4000 BCE and half a millennium later, a subgroup of them developed cultural innovations, expanded dramatically, and manifested archaeologically around 3300 BCE.

**Figure 5:**
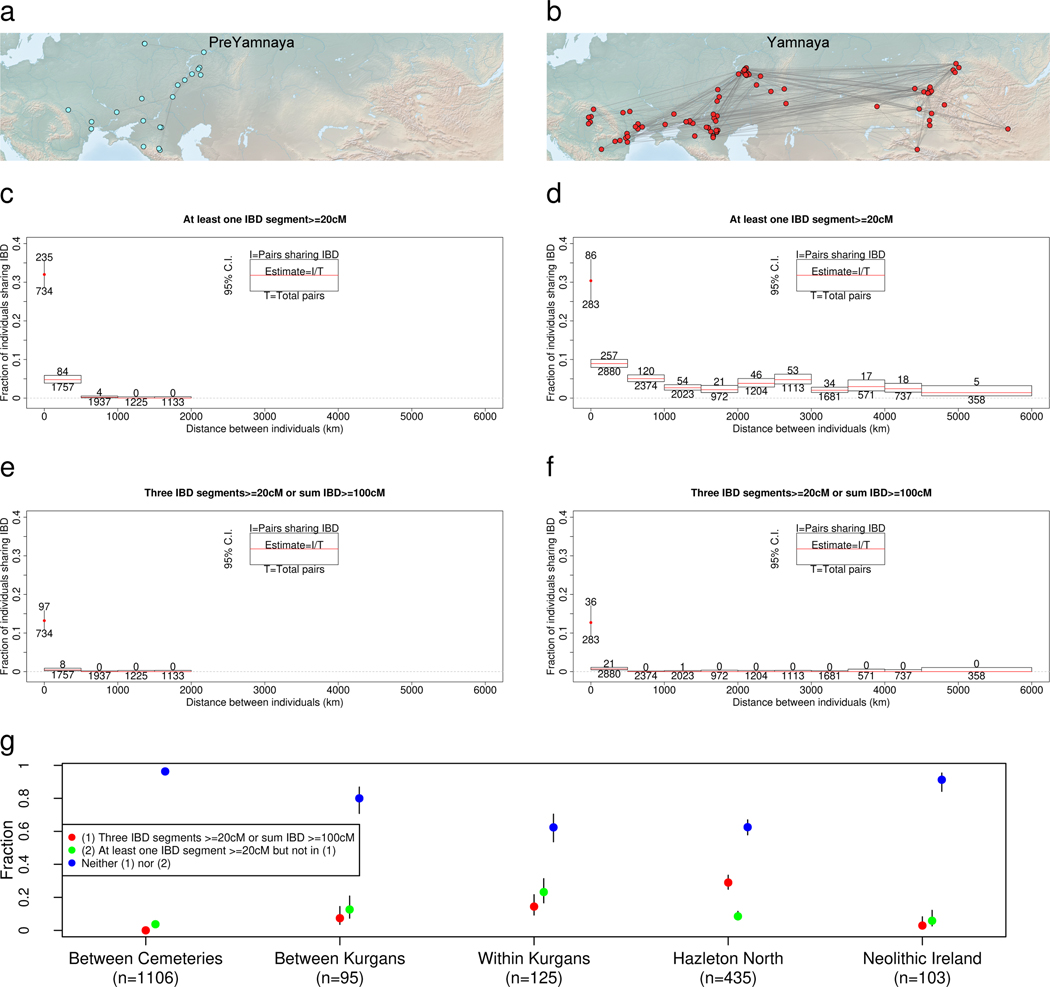
IBD analysis of the Yamnaya and their predecessors. Pairs of individuals linked by at least one IBD segment ≥20cM in length reveal a sparse but highly connected network in the Pre-Yamnaya (Methods) (a) and Yamnaya (b) groups. No detectible IBD is found in the Pre-Yamnaya period beyond the scale of 1000km (c); Yamnaya share more IBD with each other at short distance scales but IBD sharing extends all the way to the ~6000km scale of their geographical distribution (d). However, closely related individuals only occur at short distance scales in both Pre-Yamnaya (e) and Yamnaya (f) groups, indicating that the IBD sharing in the Yamnaya was a legacy of their common origin. In panels (c-f) we display the two-sided 95% confidence interval as a vertical interval (at distance=0) or rectangle (at distance ranges>0) and in red the fraction of dividing the number of pairs of individuals sharing IBD (I)/total number of pairs of individuals (T). (g) In a set of 9 Yamnaya cemeteries, and a total of 25 kurgans, closely or distantly related individuals are virtually absent in inter-cemetery comparisons, more are found in inter-kurgan/within-cemetery comparisons, and more still in intra-kurgan comparisons; nonetheless, most Yamnaya individuals in all comparisons were unrelated. Kurgan burial of close kin was less common than in the case of a local patrilineal dynasty as at a Neolithic long cairn at Neolithic Hazleton North,^46^ but more common than in Neolithic monuments of Ireland.^55^ Two-sided 95% confidence intervals are shown.

Identical-By-Descent (IBD)^44^ genomic segments ≥20cM between pairs of individuals did exist before the Yamnaya between regional populations (Fig. 5a), but these expanded dramatically in the Yamnaya period (Fig. 5b). Segments shared >500km were extremely rare pre-Yamnaya (Fig. 5c) but a few percent between 500–5000km (Fig. 6d) in the Yamnaya period. Close genetic relatives, sharing at least three ≥20cM segments (about the 5th degree)^44^, or a sum of IBD ≥100cM, were within 500km in both periods, and at a greatly elevated rate within each cemetery (Fig. 5e, f). Around 14.4% of Yamnaya-Afanasievo individual pairs within kurgans were close relatives and 7.4% of them across kurgans of the same cemetery, much lower than the 29.0% in the tightly connected pedigree of the Hazleton North chambered tomb in Neolithic Britain ~3700 BCE^46^ (p=0.00075; Fisher’s exact test). Kurgans were thus not “family tombs”^47^ of biological relatives; biological kinship in them was mostly due to common descent centuries in the past and any close kinship links within kurgans were largely non-biological.

#### Origin of Indo-Anatolian languages

A traditional view defines “Indo-European” (IE) to include Anatolian languages as the first split^48,49^. We use here a newer terminology that denotes the entire group “Indo-Anatolian” (IA) and restricts IE to the related non-Anatolian language families including Tocharian, Greek, and Sanskrit.^4,10^ The split of IA is linguistically dated to 4300–3500 BCE ^4,39,49,50^ predating both the attestation of the Hittite language in Central Anatolia (post-2000 BCE^4^) and the Yamnaya expansion. We identify the Yamnaya as Proto-IE for several reasons. Their ~4000BCE formation and mid-4^th^ millennium BCE expansion correspond to the IE-Anatolian split; they drove the Afanasievo migration^12^, plausibly carrying languages ancestral to Tocharian, widely recognized as the second, post-Anatolian, split;^51^ they are linked post-2500 BCE to Armenians and to the Balkans^3^ where, Greek, and lesser known IE languages such as Illyrian and Thracian were spoken;^10,36^ they are linked indirectly to IE speakers of central-northern Europe via the transformative Corded Ware^2,12^ and Beaker^7^ derivative cultures of the 3^rd^ millennium BCE; finally, via Fatyanovo^52^ and Sintashta^8,34^ Corded Ware descendants, also to Indo-Iranians.

**Extended Data Figure 5:**
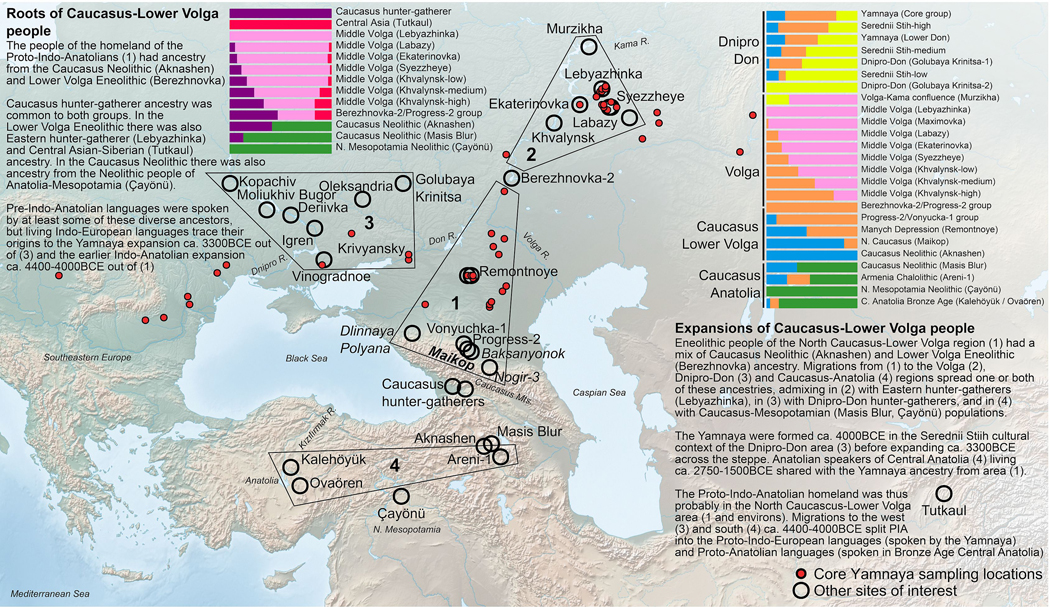
The origin of Indo-Anatolian and Indo-European languages. Genetic reconstruction of the ancestry of Pontic-Caspian steppe and West Asian populations points to the North Caucasus-Lower Volga area as the homeland of Indo-Anatolian languages and to the Serednii Stih archaeological culture of the Dnipro-Don area as the homeland of Indo-European languages. The Caucasus-Lower Volga people had diverse distal roots, estimated using the *qpAdm* software on the left barplot, as Caucasus hunter-gatherer (purple), Central Asian (red), Eastern hunter-gatherer (pink), and West Asian Neolithic (green). Caucasus-Lower Volga expansions, estimated using *qpAdm* on the right barplot, disseminated Caucasus Neolithic (blue)-Lower Volga Eneolithic (orange) proximal ancestries, mixing with the inhabitants of the North Pontic region (yellow), Volga region (yellow), and West Asia (green).

Yamnaya and Anatolians share CLV ancestry (Fig. 2e,f) which must stem from Proto-IA language speakers—save for the possibility of an early transfer of language without admixture. That the CLV ancestry in Central Anatolians during the Hittite presence included Lower Volga-related ancestry implies an origin north of the Caucasus (Fig. 2f; Extended Data Fig. 1). Long (≥30cM) IBD segments shared by Igren-8 Serednii Stih and Areni-1 with Berezhnovka-2 document Eneolithic links of the Lower Volga ancestry (Extended Data Table 5), and one link (15.2cM) between the North Caucasus Vonyucka-1 with early Bronze Age Ovaören (MA2213) ties Central Anatolia to this once expansive network. Yet, only two IA descendant groups transmitted their languages to posterity: the Yamnaya, aided by their horse-wagon technology,^6^ and the Anatolian speakers, surviving long enough for their languages to be recorded around 2000 BCE,^5^ vanishing in Late Antiquity, and fortuitously rediscovered in the 20^th^ century. Our reconstruction based on genetics (Extended Data Fig. 5) has traced both groups to the CLV people north of the Caucasus, but cannot discern who first spoke pre-IA languages.

Linguistic evidence has been advanced in favor of different solutions of the Proto-IE origins problem for more than two centuries and we review some recent proposals relevant to our reconstruction of early IA/IE history.

First, cereal terminology in IA/IE languages may restrict IA origins to the easternmost extent of agricultural subsistence during the Eneolithic, the Dnipro Valley.^53^ Our findings do not contradict this, but raise the possibility of a Caucasus (rather than European) Neolithic source for this vocabulary via the CLV Cline.

Second, the attestation of Anatolian languages largely in central-western Anatolia can most parsimoniously be explained by a western entry (via the Balkans),^4^ but genetic data provide strong evidence in favor of an eastern route^54^ as not only CLV but especially Mesopotamian Neolithic, the two sources of the Central Anatolian Bronze Age groups, are eastern. Further evidence comes from observing no European farmer or hunter-gatherer ancestry in Central Anatolian Bronze Age groups as might be expected from a Balkan route from the west (although if these groups bypassed local Europeans, or used a maritime route, we would not see European mixture). A weakness of the eastern entry hypothesis has always been that there is no linguistic evidence of Anatolian speakers in eastern Anatolia along the proposed migratory path. However, this argument does not add relative weight to the western entry hypothesis either as no linguistic evidence for migratory Pre-Anatolian speakers is to be found in the Southeastern European path proposed by that hypothesis. The lack of linguistic traces in Eastern Anatolia could be explained by the archaeologically momentous expansion of the Kura-Araxes archaeological culture in the Caucasus and eastern Anatolia after around 3000 BCE, which may have driven a wedge between steppe and West Asian speakers of IA languages, isolating them from each other and perhaps explaining their survival in western Anatolia into recorded history. That the expansion of the Kura-Araxes archaeological culture could have had a profound enough demographic impact to have pushed out Anatolian-speakers, is in fact directly attested by genetic evidence showing that in Armenia, the spread of the Kura-Araxes culture was accompanied by the complete disappearance of CLV ancestry that had appeared there in the Chalcolithic (Fig. 2f).^10,13^

The Kura-Araxes culture may not be the only reason for the IA split. Autosomal and Y-chromosome homogenization of the Yamnaya ancestral population in the 4^th^ millennium BCE provides another lens through which to understand its origins, with isolation fostering linguistic divergence. This may have persisted post-expansion: previous inhabitants largely disappear in the face of the Yamnaya juggernaut, albeit with exceptions^17^. Did mixing, avoided by the kurgan elites, occur between locals and Yamnaya not buried in kurgans? The rise of the Yamnaya on the steppe at the expense of their predecessors was followed by their demise after a thousand years, displaced by descendants of people of the Corded Ware culture. Was this the fall of the kurgan elites or the population as a whole? The steppe was dominated by many and diverse groups later still, such as the Scythians and Sarmatian nomads of the Iron Age. These groups were certainly very diverse genetically, but their kurgans scattered across the steppe attest to the persistence of at least some elements of culture that began in the Caucasus-Volga area seven thousand years ago before blooming, in the Dnipro-Don area, into the Yamnaya culture that first united the steppe and impacted most of Eurasia. To what symbolic purpose did the Yamnaya and their precursors erect these mounds we may never fully know. If they aimed to preserve the memory of those buried under them, they did achieve their goal, as the kurgans, dotting the landscape of the Eurasian steppe, drew generations of archaeologists and anthropologists to their study, and enabled the genetic reconstruction of their makers’ origins presented here.

## Methods

### Terminology for archaeological cultures and geographic locations:

For archaeological cultures and geographic locations that span more than one modern country, we used the prevalent term in the archaeological and genetic literature, for example “Yamnaya” which is the common term in Russia and most of Eastern Europe instead of the Ukrainian “Yamna”. For archaeological cultures and locations that are confined to a single country, we generally use the local terminology, for example we refer to the archaeological cultures of “Usatove” and “Trypillia” and “Serednii Stih” and the river “Dnipro” with the Ukrainian terms rather than the corresponding Russian terms “Usatovo”, “Tripolye,” “Sredni Stog” and “Dniepr”.

### Previously published Eneolithic and Yamnaya/Afanasievo individuals:

We counted previously published Yamnaya/Afanasievo individuals with genome-wide autosomal data (*n*=75) from the archaeogenetic literature.^2,3,8–10,12,34,56–62^ We counted pre-Yamnaya Eneolithic individuals^3,9,11,17,20,42,52,63,64^ with genome-wide data from the European steppe and its environs (*n*=82) by filtering individuals to the 5000–3500 BCE date range, the countries of Russia and Ukraine, and latitude west or equal to 60E and longitude south or equal to 60N.

### Sampling ancient individuals:

The skeletal remains analyzed here were all analyzed with permission from local authorities in each location from which they came. Every sample is represented by stewards such as archaeologists or museum curators, who are either authors or thanked in the Acknowledgments. The remains were almost all sampled in ancient DNA clean rooms either at Harvard Medical School, the University of Vienna, or the Institute for Archaeogenomics in Budapest. If available and accessible, we prioritized sampling petrous bones, taking bone powder from the cochlea by sandblasting and milling^65^, or directly drilling into the cochlea after physical surface cleaning, or drilling through the cranial base to minimize damage to intact skulls^66^. If we could not sample from the cochlea, we sought to sample a tooth, prioritizing the cementum layer after physical surface cleaning^67^. If neither a cochlea nor a tooth was available, we sought to sample a dense cortical bone, which we analyzed by drilling and collecting powder after physical surface cleaning. For some samples that could not leave the museum, we sampled on site, either drilling directly into the cochlea, the tooth root, or bone after physical surface removal. We sometimes dislodged auditory ossicles during sandblasting or drilling into the cochlea. When this happened during the cleaning procedure, we generally stopped the destructive sampling and collected the ossicle(s)^68^. As suggested in the study that recognized the high preservation of DNA in ossicles, we cleaned the ossicle with 10% bleach and radiated it with ultraviolet light for 10 minutes before submerging it in extraction buffer without attempting to produce powder.

### Ancient DNA data generation:

The samples for which we report new data were processed between 2013 and 2023 and therefore were analyzed with an evolving set of protocols. Details and protocols used for each library can be found in Online Table 2. At Harvard Medical School, where the majority of wet laboratory work was done, we initially carried out all DNA extractions and Illumina library preparations manually, using small batches of samples and silica columns for DNA cleanup^69–71^. Beginning in 2018, we used automated liquid handlers (Agilent Bravo Workstations) for both DNA extraction^72^ and library preparation with magnetic beads (see supplementary material in ^73^ for automated double-stranded library preparation, and ref. ^74^ for automated single-stranded library preparation). We treated DNA extracts with USER (NEB) during library preparation to cut DNA at uracils; this treatment is inefficient at terminal uracils and leaves a damage pattern expected for ancient DNA at the terminal bases that can be filtered out for downstream analysis while allowing a library to be authenticated as old. All libraries were either dual barcoded through double-stranded ligation or dual indexed through indexing PCR at the end of single-stranded library preparation to allow pooling before sequencing.

Before 2015, we screened libraries for mitochondrial DNA before attempting to capture nuclear loci^75^. In the following couple of years, we added an increasing number of nuclear SNPs (between 10 and 4000) as targets into the screening capture since mitochondrial DNA quality does not always correlate well with nuclear DNA quality and quantity. We later increased the number of targeted SNPs in our nuclear capture from about 390,000 (390k) ^2,76^ to about 1.24 million (1240k)^77^ for libraries passing the mitochondrial capture with nuclear spike-in. Later, we dropped the screening capture altogether and added the mitochondrial probes to the 1240k probes (1240k+). In 2022, we switched from the 1240k homebrew capture to a kitted capture product available from Twist Biosciences^78^.

For ancient DNA data generated in the Budapest at the Institute of Archaeogenomics, HUN-REN Research Centre for the Humanities, we followed the protocol described in ^79^.

### Bioinformatic processing:

All ancient DNA libraries were sequenced with paired-end reads. We then performed the following steps: preprocessing, alignment and post-alignment filtering for variant calling. The goal of preprocessing is to take raw sequenced products and create merged sequences for alignment. We demultiplexed reads, binned these to whichever library each read belongs to using the identifying barcodes and indices, trimmed these identifying markers as well as any residual adapter sequences, and merged each paired-end read into a single molecule using the overlap of the paired-end reads as a guide, employing a modified version of *SeqPrep* (https://github.com/jstjohn/SeqPrep ). We aligned the resulting single-ended reads to both the *hg19* human genome reference (https://www.internationalgenome.org/category/grch37/ ) and the inferred ancestral Reconstructed Sapiens Reference Sequence (RSRS) mitochondrial sequence^80^ using the *samse* aligner of *bwa*^81^. We marked duplicate molecules by barcode bin, based on the same start/stop positions and orientation. The computational pipelines with specific parameters used are publicly available on GitHub at https://github.com/dReichLab/ADNA-Tools and https://github.com/dReichLab/adna-workflow.

We used a ‘pseudohaploid genotyping’ approach to determine a randomly selected allele at SNP sets of interest. To represent the allele at each SNP, we randomly selected sequences from a pool of all sequences covering that position with a minimum data quality; our criteria were a minimum mapping quality of at least 10, and a base quality of at least 20, after trimming sequences by 2 base pairs at both the 5’ and 3’ ends to remove damage artifacts. We assessed ancient DNA authenticity by using *contamMix-1.0.1051*^82^ to search for heterogeneity in mitochondrial DNA sequences which are expected to be non-variable in uncontaminated individuals, and also ANGSD to test for heterogeneity in X chromosome sequences which are expected to be homozygous in males.^83^ We further evaluated authenticity of the ancient samples by using *pmdtools*^84^ to measure the rate of cytosine-to-thymine mutations in the first and last nucleotides (in untrimmed sequences) which is expected for genuine ancient DNA^70^, and by computing the ratio of Y chromosome to the sum of X and Y chromosome sequences which is expected to be very low for females and to have a much higher value for males. We determined a consensus for mitochondrial DNA using *bcftools* (https://github.com/samtools/bcftools ) and *SAMTools^85^*, requiring a minimum of 2-fold coverage to call the nucleotide and a majority rule to determine its value. We used *HaploGrep2* to determine mitochondrial haplogroups based on the phylotree database (mtDNA tree build 17).^86,87^

### Principal Components Analysis:

We projected individuals in Fig. 1b in *smartpca*^88^ using parameters newshrink: YES and lsqporject: YES on a PCA space whose axes are formed by the following populations: OberkasselCluster (set of trans-Alpine WHG individuals identified in^20^), Russia_Firsovo_N, Iran_HajjiFiruz_C^8^, Iran_C_SehGabi^13^, Iran_C_TepeHissar^89^, Israel_C^90^, Germany_EN_LBK^2,42,79,91^ The coordinates of plotted points are shown in Online Table 5.

### F_ST_ estimation:

We computed F_ST_ in *smartpca*^88^ with parameters inbreed: YES and fstonly: YES.^92^

### Drawing of maps:

We drew the maps in Fig. 1, Fig. 5, Extended Data Figs. 1, 5, and Supplementary Information section 2 using public domain Natural Earth data with the rnaturalearth package in R.^93^ Digital elevation maps in Supplementary Information section 1 were drawn using the Copernicus Digital Elevation Model (https://doi.org/10.5270/ESA-c5d3d65 ).

### Visualizing the three Eneolithic Clines and preceding populations:

We fit models for Eneolithic cline populations (Fig. 1c) using qpAdm^2^ and with the following set of Right populations: OldAfrica, Russia_AfontovaGora3, CHG, Iran_GanjDareh_N, Italy_Villabruna, Russia_Sidelkino.SG, and Turkey_N (Fig. 1c). Diverse ternary models of preceding, Eneolithic, and Bronze Age populations are shown in Fig. 2. Individuals plotted at the triangle edge fit (p>0.05); the simpler 2-source model is plotted for individuals with a negative coefficient from one of the three sources. The corners of each triangle represent the sources. Unplotted individuals all give fits at p<0.05 and so should be viewed as poorly described by the model.

### Model competition with qpAdm/qpWave:

We used qpWave/qpAdm methods^2,18^ to characterize relationships among diverse target and source populations from the steppe and adjacent areas (Supplementary Information section 2). We use OldAfrica, Russia_AfontovaGora3, CHG, Iran_GanjDareh_N, Italy_Villabruna, Russia_Sidelkino.SG, Turkey_N as the set of Right populations for most analyses. For analysis of Anatolians, we expanded this to OldAfrica, CHG, Iran_GanjDareh_N, Italy_Villabruna, Russia_AfontovaGora3, Russia_Sidelkino.SG, TUR_Marmara_Barcın_N, TUR_C_Boncuklu_PPN, TUR_C_Çatalhöyük_N, Natufian to gain leverage for differentiating among West Asian sources. For faster computation, we ran qpWave/qpAdm on precomputed output from qpfstats runs (https://github.com/DReichLab/AdmixTools/blob/master/qpfs.pdf ) with poplistname that includes Han.DG, and all target, source, and Right populations, and parameters allsnps: YES, inbreed: NO. We performed separate qpWave/qpAdm runs directly on genotype files as needed when the target or source populations were not present in the qpfstats output with parameter basepop: Han.DG. We identified feasible models as having p>0.05, all standard errors ≤0.1, and admixture proportions ≤2 standard errors from 0 and 1. We removed target or source populations from the Right set. Competition of models A and B involves two qpWave/qpAdm runs in which all sources of A \ B and B \ A (\ denotes set difference) are placed on the Right set. Details of all analyses can be found in Supplementary Information section 2.

### Y-chromosome haplogroup inference:

We used the methodology described in ref. ^6^ which used the YFull YTree v. 8.09 phylogeny (https://github.com/YFullTeam/YTree/blob/master/ytree/tree_8.09.0.json ) to denote Y-chromosome haplogroups in terminal notation.^94^

### Estimates of dates of admixture:

We used DATES^8,43^ to estimate dates of admixture for the Core Yamnaya, Don Yamnaya, Eastern European Yamnaya, Corded Ware, and Caucasus-Anatolian populations (Extended Data Fig. 2). For the Core Yamnaya and Caucasus-Anatolian populations, we used sets of diverse West Asian and European hunter-gatherer populations as the two sources. For the Don Yamnaya we used the Core Yamnaya and UNHG as the two sources. For the Eastern European Yamnaya we used the Core Yamnaya and a diverse set of Neolithic/Chalcolithic “European farmers” from Extended Data Fig. 3b. For the Corded Ware we used the Core Yamnaya and Globular Amphora as the two sources. It is more important to use many source samples even if they are genetically somewhat drifted to the true ones; picking the wrong sources does not bias the date estimate^43^.

### Identity-by-Descent (IBD) segment detection:

We used ancIBD^44^ to detect IBD segments of length ≥8cM. Pre-Yamnaya individuals plotted in Fig. 5 are from the 5500–3500 BCE period.

### Geographical distance estimation:

To study the decay of IBD with geographical distance, we estimate distance between sites based on their latitude and longitude given in Online Table 4, using the Haversine distance as implemented in distHaversine^95^ of the package *geosphere* in R.

### Estimates of effective population sizes:

We ran HapNe-LD (version 1.20230726 ^18^) using default parameters and providing pseudo-haploid genotypes as input. Briefly, HapNe-LD uses a summary statistic measuring long-range correlations between markers to infer fluctuations in effective population size (defined as the inverse of the coalescence rate) through time. We studied two distinct sets of unrelated individuals all of which had a coverage of at least 0.7x on the target autosomal SNPs and with a standard deviation on their estimated date smaller than 180 years (~6 generations). The first group consists of 25 Core Yamnaya individuals with estimated dates ranging between 4500 and 4800 BP. The second group contains 26 Core Yamnaya individuals ranging from 4800 to 5100 BP.

If no evidence of effective population size fluctuations can be detected in the data, HapNe-LD produces a flat line. An output containing fluctuations should thus be interpreted as the detection of changes in historical effective population sizes. Recent admixture between highly differentiated populations (Fst > 0.1) might lead to biases in LD-based analyses that induce fluctuations similar to a population bottleneck. However, HapNe implements a test to flag the presence of recent structure in the data, which was not detected in both sample sets (approximate p>=0.1), suggesting that the observed signal instead reflects variation in the effective population size of these groups.

In our analyses, the effective population size is defined as the inverse of the instantaneous coalescence rate. This quantity corresponds to twice the number of breeding individuals in an idealized population. In addition to changes in the number of individuals in the population (census size), several factors, such as changes in population structure, selection, and cultural practices,^96^ can have an influence on the effective population size. These additional factors may in part be responsible for the effective size fluctuations observed in the Core Yamnaya.

We inferred approximate confidence intervals using bootstrap with different chromosome arms as resampling units. We determined the beginning of the expansion by using the location of the minimum of each bootstrapped trajectory. We converted the results into years by assuming 28.6 years per generation for the median minimum location and 25.6 and 31.5 years per generation for the lower and upper bounds, respectively.^97^ We used these values, corresponding to the estimated number of years per generation for males (31.5) and females (25.6) to account for uncertainty in the conversion factor.

### Data Access

Genotype data for individuals included in this study can be obtained from the Harvard Dataverse repository through the following link (https://doi.org/10.7910/DVN/QGNMRH ). The DNA sequences reported in this paper are deposited in the European Nucleotide Archive under accession number PRJEB81467. Other newly reported data such as radiocarbon dates and archaeological context information are included in the manuscript and supplementary files.

## Extended Data

**Extended Data Table 1:** F_ST_ values among select populations of the Dnipro, Don, Volga, and Caucasus areas. F_ST_ values are shown below the diagonal and their standard errors above it.

	BPgroup	CoreYamnaya	Ekaterinoka	GK1	Khi	KhlokovBugor	Klo	Kmed	Labazy	Maikop	Maximovka	Murzikha	PVgroup	Remontnoye	Russia_Caucasus_LateMaikop	Russia_Don_EBA_Yamnaya	SShi	SSmed	Syezzheye	Ukraine_N	Urakozovskaya	UpperVolga
BPgroup		0.001	0.001	0.003	0.001	0.002	0.001	0.001	0.002	0.001	0.002	0.001	0.002	0.002	0.005	0.001	0.001	0.001	0.003	0.001	0.003	0.001
CoreYamnaya	0.011		0.000	0.003	0.000	0.002	0.000	0.001	0.002	0.001	0.002	0.001	0.002	0.001	0.004	0.000	0.001	0.001	0.002	0.001	0.002	0.000
Ekaterinovka	0.030	0.032		0.003	0.001	0.002	0.000	0.001	0.002	0.001	0.002	0.001	0.002	0.002	0.004	0.000	0.001	0.001	0.003	0.001	0.003	0.000
GK1	0.042	0.041	0.045		0.003	0.007	0.003	0.003	0.005	0.004	0.006	0.003	0.005	0.005	0.018	0.003	0.004	0.005	0.009	0.003	0.006	0.003
Khi	0.007	0.014	0.019	0.039		0.002	0.001	0.001	0.002	0.001	0.002	0.001	0.002	0.002	0.004	0.001	0.001	0.001	0.003	0.001	0.003	0.001
KhlopkovBugor	0.010	0.017	0.022	0.037	0.008		0.002	0.002	0.003	0.003	0.003	0.002	0.003	0.003	0.009	0.002	0.003	0.003	0.005	0.002	0.004	0.002
Klo	0.018	0.022	0.008	0.041	0.009	0.013		0.001	0.002	0.001	0.002	0.001	0.002	0.002	0.004	0.001	0.001	0.001	0.003	0.001	0.003	0.001
Kmed	0.014	0.018	0.015	0.042	0.006	-0.002	0.002		0.002	0.001	0.002	0.001	0.002	0.002	0.005	0.001	0.001	0.001	0.003	0.001	0.003	0.001
Labazy	0.032	0.034	0.009	0.048	0.021	0.027	0.010	0.016		0.002	0.003	0.002	0.003	0.003	0.007	0.002	0.002	0.003	0.004	0.002	0.004	0.002
Maikop	0.031	0.025	0.064	0.064	0.037	0.043	0.052	0.045	0.067		0.003	0.001	0.002	0.002	0.006	0.001	0.002	0.002	0.003	0.001	0.003	0.001
Maximovka	0.044	0.041	0.021	0.048	0.033	0.033	0.021	0.028	0.021	0.076		0.002	0.003	0.003	0.007	0.002	0.003	0.003	0.004	0.002	0.003	0.002
Murzikha	0.056	0.053	0.034	0.065	0.044	0.047	0.034	0.039	0.034	0.088	0.018		0.002	0.002	0.004	0.001	0.001	0.001	0.003	0.001	0.003	0.001
PVgroup	-0.002	0.012	0.035	0.046	0.010	0.012	0.024	0.018	0.038	0.025	0.048	0.061		0.003	0.006	0.002	0.002	0.003	0.004	0.002	0.003	0.002
Remontnoye	0.012	0.011	0.040	0.041	0.015	0.020	0.028	0.024	0.046	0.012	0.052	0.065	0.011		0.006	0.002	0.002	0.002	0.004	0.002	0.003	0.002
Russia_Caucasus_LateMaikop	0.025	0.020	0.058	0.065	0.033	0.037	0.048	0.041	0.059	-0.001	0.063	0.081	0.026	0.002		0.004	0.006	0.007	0.011	0.004	0.007	0.004
Russia_Don_EBA_Yamnaya	0.014	0.005	0.029	0.040	0.014	0.019	0.019	0.018	0.030	0.030	0.037	0.048	0.016	0.015	0.025		0.001	0.001	0.003	0.001	0.003	0.001
SShi	0.009	0.011	0.027	0.034	0.013	0.014	0.017	0.017	0.029	0.030	0.036	0.048	0.010	0.016	0.034	0.011		0.002	0.004	0.001	0.003	0.001
SSmed	0.011	0.010	0.021	0.034	0.011	0.012	0.015	0.015	0.021	0.030	0.030	0.041	0.013	0.014	0.019	0.008	0.004		0.004	0.001	0.003	0.001
Syezzheye	0.045	0.047	0.022	0.059	0.034	0.035	0.026	0.033	0.029	0.082	0.043	0.050	0.049	0.056	0.077	0.042	0.040	0.034		0.003	0.004	0.003
Ukraine_N	0.046	0.039	0.036	0.047	0.040	0.042	0.032	0.037	0.036	0.063	0.038	0.048	0.049	0.049	0.055	0.029	0.031	0.017	0.055		0.003	0.001
Unakozovskaya	0.059	0.057	0.094	0.090	0.068	0.069	0.083	0.076	0.096	0.034	0.107	0.117	0.058	0.039	0.030	0.060	0.062	0.061	0.107	0.092		0.003
UpperVolga	0.044	0.040	0.021	0.048	0.033	0.035	0.019	0.028	0.019	0.073	0.015	0.027	0.049	0.051	0.067	0.033	0.035	0.026	0.038	0.029	0.103	

**Extended Data Table 2:** Extraordinary genetic homogeneity in the Core Yamnaya. We tested all populations and individuals for cladality with Samara Yamnaya. We list populations for which this is not rejected (qpWave p>0.05) and populations that include individuals that fit Core Yamnaya selection criteria (qpWave p>0.2, at least 300k SNPs, and Yamnaya or Afanasievo culture).

Population	P-value Included in Core Yamnaya		Total individuals
Populations that are a clade with Samara Yamnaya			
China Xinjiang G218 BA Afanasievo oWestEurasia	9.7E-01	1	1
Russia Chelyabinsk EBA Yamnaya	9.5E-01	5	5
Russia Volgograd EBA Yamnaya	9.0E-01	3	5
Russia Ural EBA Yamnaya contam	8.2E-01	0	1
Usatove EBA	7.9E-01	0	1
Russia Ural EBA Yamnaya	7.3E-01	5	7
Russia Afanasievo Yenisei	6.7E-01	1	1
Russia MBA Poltavka	6.5E-01	0	6
Romania EBA Catacomb	6.3E-01	0	2
Russia Orlovka EBA Yamnaya	5.5E-01	1	1
Ukraine MBA	5.1E-01	0	1
Russia Samara EBA Yamnaya possible	5.0E-01	0	1
Kazakhstan EBA Yamnaya.SG	4.8E-01	1	1
Ukraine EBA Yamnaya contam	4.4E-01	0	1
Russia LowerVolga EBA Yamnaya	3.9E-01	0	1
Moldova Crasnoe Eneolithic	3.9E-01	0	1
Russia EBA 0I.SG	3.6E-01	0	1
Ukraine EBA Catacomb	3.5E-01	0	2
Ukraine MBA Catacomb o1	3.4E-01	0	1
Moldova Eneolithic	3.3E-01	0	1
Russia BA WestManych Catacomb	3.2E-01	0	1
Mongolia Chalcolithic Afanasievo 1	2.9E-01	0	1
Russia Kalmykia EBA	2.8E-01	0	1
Russia Afanasievo.SG	2.8E-01	0	2
Russia UpperOb Eneolithic Afanasievo	2.8E-01	6	6
Russia Volgograd EBA Yamnaya 0	2.8E-01	0	1
Russia Ishkinovka EBA Yamnaya	2.5E-01	1	1
Usatove Yamnaya	2.1E-01	0	1
Latvia LN CordedWare	1.7E-01	0	1
Hungary EBA Yamnaya 1 drei. 13510 contam	1.7E-01	0	1
Brillenhohle.pmd	1.6E-01	0	1
Russia Steppe Catacomb	1.6E-01	0	4
Russia Volga EBA Yamnaya	1.3E-01	4	5
Russia Kalmykia EasternManych EMBA	1.3E-01	0	2
Russia N BA possible	1.2E-01	0	1
Russia Afanasievo	1.2E-01	18	29
Moldova Eneolithic Suvorove	1.1E-01	0	1
Russia Afanasievo Khakassia possible	9.0E-02	0	1
BOY EBA	8.8E-02	0	5
Russia Rostov Steppe NorthCaucasus BA	7.4E-02	0	1
Russia LowerDon EBA Yamnaya	6.6E-02	0	1
Moldova EBA Yamnaya	6.5E-02	4	16
Ukraine EBA Catacomb.SG	6.2E-02	0	1
Russia Afanasievo contam	5.8E-02	0	2
Romania Brailita EBA Yamnaya	5.8E-02	0	1
Slovakia EBA Yamnaya	5.1E-02	0	2
Ukraine EBA Yamnaya	5.1E-02	4	9
**Populations that are not a clade with Samara Yamnaya but include at least one individual that is**			
Romania EBA Yamnaya	3.9E-02	2	8
Russia Remontnoye EBA Yamnaya	3.5E-02	5	6
Russia Kalmykia EBA Yamnaya.SG	1.8E-02	2	6
Russia Caucasus EBA Yamnaya	1.6E-02	1	3
Hungary EBA Yamnaya	1.5E-04	1	5
Russia Caspianlnland EBA Yamnaya	1.2E-04	12	26
Russia UpperYenisey Eneolithic Afanasievo	2.3E-05	1	4
Russia Don EBA Yamnaya	2.8E-50	2	23

**Extended Data Table 3:** F_ST_ values among populations that include Core Yamnaya individuals. F_ST_ values are shown below the diagonal and their standard errors above it.

	Hungary_EBA_Yamnaya	Moldova_EBA_Yamnaya	Romania_EBA_Yamnaya	Russia_Afanasievo	Russia_CaspianInland_EBA_Yamnaya	Russia_Caucasus_EBA_Yamnaya	Russia_Chelyabinsk_EBA_Yamnaya	Russia_Don_EBA_Yamnaya	Russia_Kalmykia_EBA_Yamnaya.SG	Russia_Remontnoye_EBA_Yamnaya	Russia_Samara_EBA_Yamnaya	Russia_UpperOb_Eneolithic_Afanasievo	Russia_UpperYenisey_Eneolithic_Afanasievo	Russia_Ural_EBA_Yamnaya	Russia_Volga_EBA_Yamnaya	Russia_Volgograd_EBA_Yamnaya	Ukraine EBA Yamnaya
Hungary_EBA_Yamnaya		0.001	0.001	0.001	0.001	0.002	0.001	0.001	0.001	0.001	0.001	0.001	0.001	0.001	0.001	0.001	0.001
Moldova_EBA_Yamnaya	0.001		0.001	0.000	0.000	0.001	0.001	0.000	0.001	0.001	0.000	0.001	0.001	0.001	0.001	0.001	0.001
Romania_EBA_Yamnaya	0.001	0.001		0.001	0.001	0.002	0.001	0.001	0.001	0.001	0.001	0.001	0.001	0.001	0.001	0.001	0.001
Russia_Afanasievo	0.006	0.004	0.005		0.000	0.001	0.001	0.000	0.001	0.001	0.000	0.001	0.001	0.001	0.001	0.001	0.001
Russia_Caspianlnland_EBA_Yamnaya	0.004	0.003	0.002	0.006		0.001	0.001	0.000	0.001	0.001	0.000	0.001	0.001	0.001	0.001	0.001	0.001
Russia_Caucasus_EBA_Yamnaya	0.001	0.002	0.001	0.003	0.003		0.002	0.001	0.002	0.002	0.001	0.002	0.002	0.002	0.002	0.002	0.002
Russia_Chelyabinsk_EBA_Yamnaya	0.008	0.009	0.009	0.010	0.009	0.009		0.001	0.001	0.001	0.001	0.001	0.001	0.001	0.001	0.001	0.001
Russia_Don_EBA_Yamnaya	0.006	0.005	0.006	0.008	0.006	0.005	0.012		0.001	0.001	0.000	0.001	0.001	0.001	0.001	0.001	0.001
Russia_Kalmykia_EBA_Yamnaya.SG	0.007	0.005	0.004	0.005	0.001	0.004	0.011	0.007		0.001	0.001	0.001	0.001	0.001	0.001	0.001	0.001
Russia_Remontnoye_EBA_Yamnaya	0.004	0.004	0.003	0.004	0.000	0.003	0.010	0.006	-0.049		0.001	0.001	0.001	0.001	0.001	0.001	0.001
Russia_Samara_EBA_Yamnaya	0.003	0.002	0.003	0.005	0.003	0.003	0.008	0.005	0.005	0.004		0.001	0.001	0.001	0.001	0.001	0.001
Russia_UpperOb_Eneolithic_Afanasievo	0.006	0.005	0.004	0.002	0.003	0.006	0.010	0.008	0.001	0.003	0.004		0.001	0.001	0.001	0.001	0.001
Russia UpperYenisey Eneoiithic Afanasievo 0.011		0.010	0.008	0.009	0.009	0.009	0.015	0.012	0.009	0.007	0.010	0.006		0.001	0.001	0.001	0.001
Russia_Ural_EBA_Yamnaya	0.002	0.002	0.001	0.004	0.003	0.003	0.006	0.005	0.004	0.003	0.001	0.003	0.008		0.001	0.001	0.001
Russia_Volga_EBA_Yamnaya	0.004	0.004	0.003	0.005	0.005	0.005	0.007	0.007	0.008	0.007	0.003	0.007	0.011	0.003		0.001	0.001
Russia_Volgograd_EBA_Yamnaya	0.005	0.003	0.004	0.007	0.005	0.004	0.009	0.007	0.007	0.005	0.004	0.007	0.009	0.003	0.006		0.001
Ukraine EBA Yamnaya	0.003	0.001	0.001	0.004	0.002	0.002	0.008	0.005	0.003	0.003	0.002	0.004	0.009	0.001	0.004	0.004	

**Extended Data Table 4:** qpAdm models that fit non-Core Yamnaya. We use the following sources to model Yamnaya-related populations other than the Core and Don Yamnaya: CoreYamnaya, Romania_C_Bodrogkeresztur, Romania_N, Serbia_IronGates_Mesolithic, Trypillia, Ukraine_N, Usatove. The Baden individuals from Hungary represent a reburial into a kurgan^79^ and are predominantly of European farmer, not Yamnaya, ancestry. The Riltsi individual is shown with Usatove ancestry here and can also be modeled with about half Remontnoye ancestry, as the Usatove have ancestry from the CLV cline.^17^

Modeled group	A	B	P-value	A	B	S.E.
Bulgaria C ProtoYamnaya	Serbia IronGates Mesolithic	Usatove	0.062	17.5%	82.5%	2.4%
Bulqaria EBA Yamnaya	CoreYamnaya	Romania C Bodroqkeresztur	0.883	85.4%	14.6%	1.4%
Bulqaria EBA Yamnaya	CoreYamnaya	Romania N	0.682	86.7%	13.3%	1.3%
Bulqaria EBA Yamnaya	CoreYamnaya	Try pi Ilia	0.719	82.9%	17.1%	1.7%
Bulqaria EBA Yamnaya	CoreYamnaya	Usatove	0.396	66.7%	33.3%	3.3%
Bulqaria Riltsi EBA Yamnaya	Romania N	Usatove	0.079	25.3%	74.7%	5.8%
Hunqary LateC EBA Baden Yamnaya	CoreYamnaya	Romania C Bodroqkeresztur	0.113	3.4%	96.6%	1.5%
Hungary LateC EBA Baden Yamnaya	Romania C Bodroqkeresztur	Serbia IronGates Mesolithic	0.309	95.9%	4.1%	1.4%
Hunqary LateC EBA Baden Yamnaya	Romania C Bodrogkeresztur	Trypillia	0.114	77.6%	22.4%	9.7%
Hunqary LateC EBA Baden Yamnaya	Romania C Bodroqkeresztur	Ukraine N	0.197	96.2%	3.8%	1.5%
Hunqary LateC EBA Baden Yamnaya	Romania C Bodroqkeresztur	Usatove	0.099	94.2%	5.8%	2.5%
Hunqary LateC EBA Baden Yamnaya	Romania N	Serbia IronGates Mesolithic	0.323	87.0%	13.0%	1.7%
Hunqary LateC EBA Baden Yamnaya	Romania N	Ukraine N	0.094	86.9%	13.1%	1.7%
Moldova EBA Yamnaya	CoreYamnaya	Romania C Bodroqkeresztur	0.724	93.8%	6.2%	0.9%
Moldova EBA Yamnaya	CoreYamnaya	Romania N	0.571	94.3%	5.7%	0.8%
Moldova EBA Yamnaya	CoreYamnaya	Trypillia	0.675	92.7%	7.3%	1.0%
Moldova EBA Yamnaya	CoreYamnaya	Usatove	0.367	86.0%	14.0%	2.2%
Moldova GlobularAmphora Yamnaya	CoreYamnaya	Trypillia	0.053	88.7%	11.3%	2.6%
Romania Brailita EBA Yamnaya	CoreYamnaya	Romania C Bodroqkeresztur	0.561	91.6%	8.4%	2.4%
Romania Brailita EBA Yamnaya	CoreYamnaya	Romania N	0.524	92.4%	7.6%	2.1%
Romania Brailita EBA Yamnaya	CoreYamnaya	Trypillia	0.601	90.1%	9.9%	2.8%
Romania Brailita EBA Yamnaya	CoreYamnaya	Usatove	0.509	81.0%	19.0%	5.6%
Romania EBA Yamnaya	CoreYamnaya	Romania N	0.096	95.8%	4.2%	1.0%
Romania EBA Yamnaya	CoreYamnaya	Usatove	0.143	89.1%	10.9%	2.5%
Serbia EBA Yamnaya	CoreYamnaya	Romania C Bodroqkeresztur	0.196	87.3%	12.7%	2.0%
Serbia EBA Yamnaya	CoreYamnaya	Romania N	0.097	88.6%	11.4%	1.9%
Serbia EBA Yamnaya	CoreYamnaya	Trypillia	0.200	85.1%	14.9%	2.3%
Ukraine EBA Yamnaya	CoreYamnaya	Romania C Bodroqkeresztur	0.561	93.3%	6.7%	1.3%
Ukraine EBA Yamnaya	CoreYamnaya	Romania N	0.481	94.0%	6.0%	1.2%
Ukraine EBA Yamnaya	CoreYamnaya	Trypillia	0.756	92.0%	8.0%	1.5%
Ukraine EBA Yamnaya	CoreYamnaya	Usatove	0.534	84.5%	15.5%	3.1%

**Extended Data Table 5:** Cross-regional shared Identity-by-Descent (IBD) segments. We list all segments≥12cM shared between individuals from two different regions defined as follows. “Dnipro cline”: CoreYamnaya, GK1, GK2, Russia_Don_EBA_Yamnaya, SShi, SSlo, SSmed, Ukraine_N. Volga River basin ancestry gradients (downriver “Volga Cline” and upriver “European Hunter-Gatherer Cline”): Ekaterinovka, Khi, KhlopkovBugor, Klo, Kmed, Labazy, Lebyazhinka_HG, Maximovka, Murzikha, Syezzheye, UpperVolga. “Caucasus-Lower Volga Eneolithic”: BPgroup, PVgroup. “CLV-South”: Remontnoye, Maikop, Unakozovskaya, Armenia_C, TUR_C_Kalehöyük_MLBA, TUR_C_Ovaören_EBA

Individual 1	Individual 2	Group 1	Group 2	Segment length (cM)
122201	11924	BPgroup	SShi	35.8
I22202	I6734	BPgroup	Khi	32.1
11634	122199	Armenia C	BPgroup	31.4
I6300 enhanced	I22202	KhlopkovBugor	BPgroup	22.0
I6406	I22200	Kmed	BPgroup	20.1
PG2004	111837	BPgroup	Khi	18.4
16301 enhanced	122199	KhlopkovBugor	BPgroup	18.2
16301 enhanced	PG2001	KhlopkovBugor	PVgroup	17.6
I28683	PG2004	Remontnoye	BPgroup	16.6
110567	I28682	Russia Caspianlnland EBA Yamnaya	Remontnoye	16.2
PG2001	I3950	PVgroup	Russia Afanasievo	15.9
PG2001	I6062	PVgroup	Ekaterinovka	15.9
122199	I8282	BPgroup	Ekaterinovka	15.8
122201	110208	BPgroup	Moldova EBA Yamnaya	15.5
11924	120188	SShi	Klo	15.4
132501	18448	Russia UpperYenisey Eneolithic Afanasievo	Murzikha	15.4
112637	I8457	Moldova EBA Yamnaya	Murzikha	15.4
132821	18449	Russia UpperOb Eneolithic Afanasievo	Murzikha	15.4
MA2213 wNonUDG.SG	VJ1001	TUR C Ovaören EBA	PVgroup	15.2
132501	I8455	Russia UpperYenisey Eneolithic Afanasievo	Murzikha	15.2
16301 enhanced	122199	KhlopkovBugor	BPgroup	14.9
18411 enhanced	I26785	UpperVolga	Russia Don EBA Yamnaya	14.9
122201	11924	BPgroup	SShi	14.8
122199	I28682	BPgroup	Remontnoye	14.8
10122	I22202	Klo	BPgroup	14.6
132501	I8454	Russia UpperYenisey Eneolithic Afanasievo	Murzikha	14.5
122199	16734	BPgroup	Khi	14.5
122201	111752	BPgroup	Russia Afanasievo	14.3
I6064	122199	Ekaterinovka	BPgroup	14.2
10122	122199	Klo	BPgroup	14.2
11634	11924	Armenia C	SShi	13.9
16301 enhanced	122201	KhlopkovBugor	BPgroup	13.9
16918	18446	Russia Volgograd EBA Yamnaya	Maximovka	13.9
I22202	I6739	BPgroup	Khi	13.9
PG2004	123651	BPgroup	Ekaterinovka	13.7
I0357	111842	Russia Samara EBA Yamnaya	Murzikha	13.7
I22202	I3952	BPgroup	Russia Afanasievo	13.7
10122	120190	Klo	Russia Samara EBA Yamnaya	13.6
18951	111842	Russia Don EBA Yamnaya	Murzikha	13.5
PG2004	I8290	BPgroup	Ekaterinovka	13.4
10231	I8456	Russia Samara EBA Yamnaya	Murzikha	13.4
125159	122199	Russia Afanasievo	BPgroup	13.3
14111	16109	Ukraine N	Klo	13.3
122199	I26787	BPgroup	Russia Don EBA Yamnaya	13.3
16301 enhanced	PG2004	KhlopkovBugor	BPgroup	12.9
18449	12105	Murzikha	Ukraine EBA Yamnaya	12.9
120189	I22200	Ekaterinovka	BPgroup	12.8
I6297	122201	Russia Orlovka EBA Yamnaya	BPgroup	12.8
I6705	I28682	Russia Samara EBA Yamnaya	Remontnoye	12.8
132821	I22200	Russia UpperOb Eneolithic Afanasievo	BPgroup	12.7
132501	18449	Russia UpperYenisey Eneolithic Afanasievo	Murzikha	12.6
122201	I6739	BPgroup	Khi	12.4
10231	I28682	Russia Samara EBA Yamnaya	Remontnoye	12.3
PG2004	I6739	BPgroup	Khi	12.3
16918	I22200	Russia Volgograd EBA Yamnaya	BPgroup	12.3
122201	I3952	BPgroup	Russia Afanasievo	12.2
I6406	11450	Kmed	Russia Samara EBA Yamnaya	12.2
122199	I5273	BPgroup	Russia Afanasievo	12.1
14114	112964	Ukraine N	UpperVolga	12.1
111838	123651	Russia Volga EBA Yamnaya	Ekaterinovka	12.0
I6907	111841	Russia Samara EBA Yamnaya	Murzikha	12.0
122201	11924	BPgroup	SShi	12.0

## Supplementary Material

Supplementary Information Combined

Online Tables
